# Antimanic Efficacy, Tolerability, and Acceptability of Clonazepam: A Systematic Review and Meta-Analysis

**DOI:** 10.3390/jcm12185801

**Published:** 2023-09-06

**Authors:** Andreas S. Lappas, Bartosz Helfer, Katarzyna Henke-Ciążyńska, Myrto T. Samara, Nikos Christodoulou

**Affiliations:** 1 Department of Psychiatry, Faculty of Medicine, University of Thessaly, 41110 Larissa, Greece; samaramyrto@gmail.com (M.T.S.); nikoschristodoulou@gmail.com (N.C.); 2 Department of Geriatric Psychiatry, Aneurin Bevan University Health Board, Newport NP20 2UB, UK; 3 Meta Research Centre, University of Wroclaw, 50-137 Wroclaw, Poland; bartosz.helfer@gmail.com (B.H.); katarzyna.henke-ciazynska@uwr.edu.pl (K.H.-C.); 4 Institute of Psychology, University of Wroclaw, 50-527 Wroclaw, Poland; 5 Medical School, University of Nottingham, Nottingham NG7 2RD, UK

**Keywords:** benzodiazepines, clonazepam, bipolar disorder, mania, systematic review, meta-analysis

## Abstract

(1) Background: The use of benzodiazepines for the treatment of acute mania remains prevalent. This systematic review and meta-analysis provides an updated assessment of Clonazepam’s antimanic efficacy, tolerability, and acceptability. (2) Methods: A systematic search of multiple databases and clinical trial registries was conducted, aiming to identify any controlled studies of Clonazepam vs. placebo or any other pharmacotherapy for the treatment of acute mania. Pairwise meta-analytic evaluations were performed. (3) Results: Six studies were included with a total number of 192 participants, all of which were randomized controlled trials. Clonazepam may be superior to a placebo in the acute phase of treatment and no different to Lithium and Haloperidol in terms of efficacy, both acutely and in the medium to long term. Clonazepam may be an acceptable and well-tolerated treatment for acute mania, especially when used as an augmentation strategy. Comparisons were underpowered, with minimal sample sizes and only one study per comparison in many cases, thus limiting the generalizability of our findings and hindering firm clinical conclusions. (4) Conclusions: Given the prevalence of benzodiazepine use in current practice, more and larger studies are urgently needed.

## 1. Introduction

Bipolar Affective Disorder (BAD) is a serious and chronic mental illness that affects approximately 1% of the general population [1]. Despite advancements in treatment and management strategies, BAD is associated with a significant reduction in life expectancy, typically resulting in a loss of 10–20 years [2,3], and a 20–30 times higher risk of suicide compared to the general population [4]. Comorbidity with other psychiatric and/or medical conditions has been estimated to be up to 90% [5], and the socio-economic costs are substantial [6]. Living with BAD presents significant challenges for the affected individual [7].

Mania most commonly occurs within the context of BAD [8]. In fact, the diagnosis of BAD type I is defined by the presence of at least one manic episode in an individual’s lifetime [9]. Mania is characterized by elevated mood, increased energy, and associated symptoms such as grandiosity and decreased need for sleep. Defined by DSM-V criteria, a manic episode must persist for at least one week, accompanied by functional decline, hospitalization, or concurrent psychotic symptoms [1]. Mania is often considered a medical emergency, due to serious risks posed to the individual and others around them as a consequence of marked psychomotor agitation and impulsivity [10,11]. These include alcohol and substance misuse [12], bankruptcy, loss of close relationships, unplanned pregnancy, as well as self-neglect, vulnerability, and exploitation, and sometimes aggressiveness and violence [13].

Effective acute mania treatments, endorsed by guidelines such as NICE, comprise antipsychotic monotherapy (e.g., Haloperidol, Olanzapine, Quetiapine, Risperidone) and mood stabilizers (e.g., Lithium, Valproate) [14]. However, prescribers must grapple with limitations and safety concerns. Valproate is contraindicated in women of childbearing age due to teratogenic risk [15] and it is associated with cognitive impairment, hepatotoxicity, and pancreatitis [1]. Lithium, an effective therapy with suicide prevention benefits, poses challenges in tolerability and acceptability due to its narrow therapeutic window, demanding monitoring as well as interactions with other prescribed medication, overdose risk, and adverse effects [1]. Antipsychotics entail safety concerns affecting both short-term tolerability (EPSEs, sedation) and long-term acceptability (cardiometabolic risks, arrhythmia) [1], aggravated by metabolic syndrome with associated higher mortality [16].

Benzodiazepines are discouraged as a first-line therapy due to dependency and cognitive risks [17]. Despite guideline non-endorsement, benzodiazepine use remains widespread in clinical practice, both during the acute phase and in the long term [17,18,19,20]. Their indisputable efficacy for rapid tranquilization [21], coupled with effectiveness in addressing insomnia [22] and agitation [18], combined with a dearth of comparable, safer, and immediately effective pharmacotherapy alternatives [21], potentially implies a clinical necessity to deviate from guidance.

Clonazepam is one of the most frequently utilized long-acting benzodiazepines for the treatment of acute mania [20]. In fact, acute mania is still recognized as one of its common off-label uses, which distinguishes it from other benzodiazepines [23]. It has been suggested that, in addition to its sedative action, Clonazepam may exert its antimanic effects through inhibition of dopamine release [24,25]. Furthermore, it has been postulated that one of the benefits of using Clonazepam for the treatment of acute mania is mitigation of extrapyramidal adverse effects of antipsychotics, as lower doses may be required when Clonazepam is prescribed as an add on to antipsychotic treatment [26].

Several studies have examined the antimanic efficacy of Clonazepam, yielding conflicting results. Chouinard [27], as well as Sachs and colleagues [28], have advocated for the antimanic efficacy of clonazepam, while Aronson and colleagues reported a lack of efficacy [29].

The evidence evaluating the antimanic efficacy, acceptability, and tolerability of Clonazepam has not been recently assessed. To the best of our knowledge, the only existing systematic review and meta-analysis attempting to evaluate this evidence is nearly 20 years old (published in 2004, searched up to 2000), and it only included four studies involving the use of Clonazepam [30]. However, despite the limitations, the review indicated that Clonazepam appeared to be an effective and safe treatment for acute mania, even when administered as a monotherapy [30].

Given the prevalent use of Clonazepam for the treatment of acute mania in clinical practice, an up-to-date systematic review and meta-analysis examining its antimanic efficacy, tolerability, and acceptability are important and may offer new clinical insights and directions for future research.

## 2. Materials and Methods

An a priori written study protocol was published in PROSPERO (number: CRD42023432231) and is presented in the Supplementary Materials (Supplement A). The review was conducted in accordance with the Preferred Reporting Items for Systematic Reviews and Meta-Analyses (PRISMA) statement (Supplement C in the Supplementary Materials) [31].

### 2.1. Population

Participants were of any gender, ethnicity, age, and in any setting, meeting the following inclusion criteria: operationalized or clinical diagnosis of an acute manic episode (any form of ICD, DSM, Feighner 1972 [32], Spitzer 1978 [33], or a clinical diagnosis as reported by the authors). This could have been in the context of a Bipolar Affective Disorder with or without psychotic symptoms, not excluding mixed affective states and rapid cycling illness. In addition, any participants with mania in a Schizoaffective disorder, or any other form of affective psychosis, were included. Lastly, any participants with mania in the context of puerperal psychosis, as well as organic mania (in the context of delirium or of another etiology, for example, steroid-induced mania, thyrotoxicosis, etc.), were not excluded.

### 2.2. Interventions and Types of Study Eligible for Inclusion

Clonazepam, used in any form or preparation (for example, oral tablets or capsules or an intramuscular injection) either as monotherapy or as augmentation of other pharmacotherapy and compared with a placebo or any other pharmacotherapy, such as a different antimanic/tranquilizing drug, given in any form or preparation was considered.

Eligible study designs included individual and cluster randomized controlled trials (RCTs), quasi-randomized trials, and non-randomized controlled trials, irrespective of the use of blinding (open-label or single- or double-blind).

### 2.3. Search Strategy and Selection Criteria

We searched Medline (via Ovid), Embase, APA PsycINFO, the Cochrane Central Register of Controlled Trials (CENTRAL), ClinicalTrials.gov, and the WHO International Clinical Trials Registry Platform (ICTRP) starting from their respective inception dates until the end of April 2023. The search strategy is available in the Supplementary Material (Supplement B). There were no restrictions on the language, date, and publication status of the studies.

At least two reviewers (A.S.L., B.H., K.H.-C.) independently screened all the abstracts and then the full texts identified from the searches. The process was facilitated by the use of Rayyan [34]. Conflicts were resolved by discussions between the reviewers, and discussions with the senior authors (M.T.S., N.C.). At least two reviewers (A.S.L., B.H., K.H.-C.) independently decided whether the studies met the inclusion criteria.

### 2.4. Outcome Measures and Data Extraction

Primary outcomes:

(i) Efficacy: response to treatment (continuous), measured as mean change scores (which were given preference) or endpoint scores (if data on change scores were not available), in symptoms of mania, using the Young Mania Rating Scale [35], any other validated rating scale, or authors’ definitions and measures of response, if the preferred data were not available.
(ii) Tolerability (dichotomous): the proportion of participants who dropped out due to treatment emergent adverse effects (between the first treatment dose and endpoint).
(iii) Acceptability (dichotomous): proportion of participants who dropped out due to any reason (all-cause discontinuation between the first treatment dose and endpoint).

Secondary outcomes:

(i) Efficacy (dichotomous): response to treatment, defined as a reduction of ≥50% in the mean YMRS (Young Mania Rating Scale, which was given preference) [36] or any other similar validated rating scale, compared to baseline, or however defined by the authors if the above data were not available.
(ii) Remission (dichotomous), measured as the proportion of patients in remission following treatment. Remission was defined as a YMRS score of ≤12 [36], equivalent on another validated scale, or however defined by the authors if the above data were not available.
(iii) Efficacy: global state (continuous): change scores (which were given preference) or endpoint scores measured by the CGI (Clinical Global Impression) scale [37] (which was given preference) or any other similar validated rating scale.
(iv) Efficacy: functioning (continuous): change score (preferable) or endpoint scores, measured by rating scales such as the Global Assessment of Functioning [38] or any other published rating scale.
(v) Efficacy: psychotic symptoms (continuous): change scores (which were given preference) or endpoint scores measured by the Brief Psychiatric Rating Scale [39] or any other validated scale.
(vi) Tolerability: specific adverse effects (continuous and/or dichotomous): proportion of participants experiencing specific treatment emergent adverse effects, change score or endpoint scores of any adverse effect rating scale. Examples of adverse effects include sedation, extrapyramidal side effects, and ataxia, among others.
(vii) Use of antipsychotic medication as required: continuous (total antipsychotic dose utilized) and/or dichotomous (proportion of patients requiring rescue antipsychotic treatment in each group).
(viii) Total dose of mood stabilizer in each group (continuous).
(ix) Suicidality (dichotomous and/or continuous), as defined by the authors.
(x) Quality of life (continuous), change score (preferable) or endpoint scores in any published rating scale (e.g., Quality of Life Scale [40]).
(xi) Relapse in the course of follow-up (dichotomous), as defined by the authors.
(xii) Insomnia (continuous): change score (preferable) or endpoint score of total nocturnal sleep time, defined as the total amount of time of sleep using subjective (for example, a sleep diary) or objective measures (for example, polysomnography).

We divided both primary and secondary outcomes into acute (up to 3 weeks [41]) and long term (over 3 weeks) outcomes.

Data extraction was performed by two reviewers independently (A.S.L., K.H.-C.) using the same spreadsheets. Conflicts were resolved through discussion with the senior authors (B.H., M.T.S., N.C.).

### 2.5. Statistical Analysis

We conducted a pairwise meta-analysis using the Review Manager 5.4 software [42] to synthesize the data. In this analysis, we employed a random-effects model, which tends to be more conservative in terms of assessing statistical significance. However, it is important to note that this approach can place greater emphasis on smaller studies, potentially leading to an overestimation or underestimation of the effect size [43]. To ensure the robustness of our findings, we conducted a sensitivity analysis for the primary outcomes, exploring the impact of using a fixed-effects model.

For all our analyses, we adopted an intention-to-treat (ITT) approach whenever feasible. When dealing with dichotomous outcomes, we favored the Odds Ratio (OR) as the effect size. For continuous outcomes, we used the weighted mean difference (MD). In cases where data were reported on different scales, we opted for the standardized mean difference (SMD). In instances where standard deviations were missing, we derived them from available information such as standard errors, confidence intervals, t-values, or *p*-values. Whenever possible, we calculated means of means along with their corresponding standard deviations (SD).

We investigated heterogeneity visually through forest plots and employed statistical tests to assess them. The χ^2^ test was used to evaluate statistical heterogeneity, while the I^2^ statistic was calculated to quantify the degree of heterogeneity along with its 95% confidence interval (CI).

To explore potential sources of heterogeneity, we planned several subgroup analyses based on predefined variables. These variables included:

1. Primary diagnosis or, if data were limited, distinguishing between mania with psychosis and mania without psychosis.
2. Rapid cycling mania versus non-rapid cycling.
3. Treatment-resistant mania versus non-treatment-resistant mania.
4. Monotherapy versus add-on drug treatment.
5. Distinct age groups: children and adolescents versus adults.
6. Adults aged 65 and older versus those younger than 65.
7. Presence of comorbid substance misuse versus no comorbidity.
8. Any other variables relevant to investigating heterogeneity.

In addition, we preplanned sensitivity analyses to assess the robustness of our results. These sensitivity analyses included:

1. Exclusion of studies that did not adhere to operationalized diagnostic criteria.
2. Exclusion of non-randomized studies.
3. Comparison of the fixed-effects model versus the random-effects model.
4. Exclusion of studies with imputed data.
5. Exclusion of sponsored studies.

### 2.6. Risk of Bias Assessment

Two review authors (A.S.L., K.H.-C.) independently assessed the risk of bias of the included studies, using the Cochrane risk of bias tool (study based) for randomized trials (RoB) [44].

## 3. Results

### 3.1. Search

We identified six studies that met our inclusion criteria. The studies were published from 1983 to 2009 and included 192 participants in total.

### 3.2. Characteristics of Included Studies

Interestingly, we did not find any non-randomized controlled trials, and all six studies identified were RCTs. The PRISMA flow diagram is shown in Figure S1 (Supplementary Materials). Table 1 presents the characteristics of all included studies. Three studies took place in Canada [45,46,47], one in New Zeeland [48], one in South Africa [49], and one in China [50]. In all studies, participants had a diagnosis of acute mania fulfilling the DSM-III, DSM-IV, or Spitzer 1978 [33] criteria. All participants were adults of working age, and no studies including children, adolescents or an older adult population were identified. All included studies took place in inpatient settings, with moderate to severe illness at baseline. No study included patients with current comorbid substance misuse, current rapid cycling BAD, or treatment resistant mania.

Only one placebo-controlled RCT was identified (N = 40, [48]). All other studies compared Clonazepam with another antimanic/tranquilizing drug, namely Lorazepam (one RCT, N = 24, [47]), Lithium (two RCTs, N = 52, [45,49]), and Haloperidol (two RCTs, N = 76, [46,50]).

Three RCTs only studied acute antimanic efficacy, with a duration of 1 day ([46], antimanic effects of rapid tranquilization), 5 days ([48], the only placebo-controlled trial) and 10 days [45]. The remaining three RCTs also examined medium- (up to 4 weeks [47,49]) and long-term effects (up to 16 weeks [50]).

Clonazepam was prescribed as an oral monotherapy in four RCTs [45,47,48,49]. In all but one of the above RCTs [47], concurrent treatment with neuroleptics as-required was permitted. In the second phase of the Bradwejn 1990 RCT (day 15 to day 28 [47]), Lithium augmentation was added to oral Clonazepam or Lorazepam. In the Young 2009 study, oral Clonazepam or oral Haloperidol were add-ons to treatment with Valproate [50]. Clonazepam was prescribed as intramuscular injection (IM) in one RCT [46] examining the antimanic efficacy of IM Clonazepam vs. IM Haloperidol when used for rapid tranquilization. A total of 75% of patients in this study were on regular antipsychotic medication at the point of entry.

### 3.3. Risk of Bias Assessment

**Figure 1:**
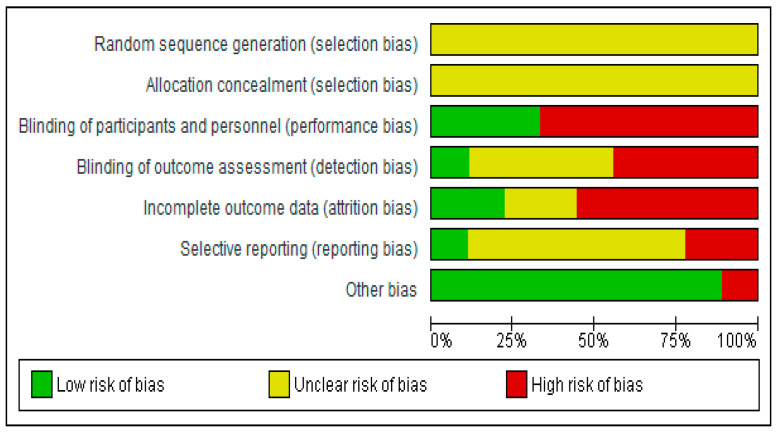
Summary of risk of bias assessment.

The results of the assessment of the risk of bias are presented in Figure 1. No study was judged to have an overall low risk of bias, and the risks of performance and attrition bias were high for more than 50% of the studies.

### 3.4. Primary Outcomes

#### 3.4.1. Efficacy: Response to Treatment

##### Clonazepam vs. Placebo

The only identified placebo-controlled study [48] demonstrated the significant superiority of Clonazepam over a placebo in terms of acute antimanic efficacy (Figure S2 in Supplementary Materials, SMD = −1.23, 95%CI = [−1.91, −0.55], *p* = 0.0004, one RCT, N = 40).

##### Clonazepam vs. Lorazepam

Data from the only study comparing Clonazepam with another benzodiazepine, namely Lorazepam [47], showed a trend for the superiority of Lorazepam over Clonazepam in terms of acute antimanic efficacy (14 days, monotherapy, SMD = 0.82, 95%CI = [−0.02, 1.67], *p* = 0.06, one RCT, N = 24). In the second phase of this study, Lithium was added to either Clonazepam or Lorazepam and the participants were followed-up for a further 14 days (28 days in total). In the medium term (28 days), no significant differences between the efficacy of Clonazepam and that of Lorazepam were identified (SMD = 0.82, 95%CI = [−0.13, 1.76], *p* = 0.09, one RCT, N = 19). The results are also presented in Figure S3.

##### Clonazepam vs. Lithium

**Figure 2:**
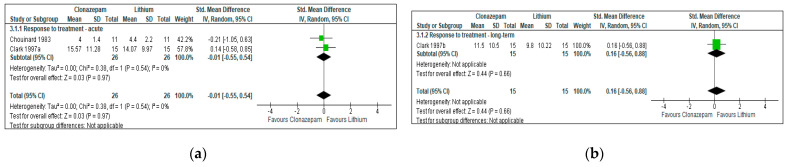
Response to treatment: Clonazepam vs. Lithium. (**a**): acute effects (up to 3 weeks). Clark 1997a [49]: endpoint: 3 weeks. (**b**): long-term effects (more than 3 weeks of continuous treatment). Clark 1997b [49]: endpoint: 4 weeks.

Two RCTs provided data [45,49]. There was no difference between Clonazepam and Lithium, both in terms of acute (up to 3 weeks, SMD = −0.01, 95%CI = [−0.55, 0.54], *p* = 0.54, two RCTs, N = 52) and medium-term (4 weeks) antimanic efficacy (SMD = 0.16, 95%CI = [−0.56, 0.88], *p* = 0.66, one RCT, N = 30, Figure 2 and Figure S4).

##### Clonazepam vs. Haloperidol

**Figure 3:**
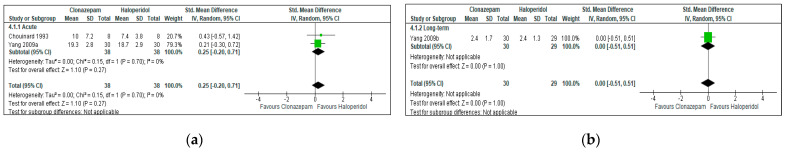
Response to treatment: Clonazepam vs. Haloperidol. (**a**): acute effects (up to 3 weeks). Yang 2009a [50]: endpoint: 3 weeks. (**b**): long-term effects (more than 3 weeks of continuous treatment). Yang 2009b [50]: endpoint: 16 weeks.

Two RCTs provided data [46,50]. There was no difference between Clonazepam and Haloperidol, both in terms of acute (up to 2 weeks, SMD = 0.25, 95%CI = [−0.20, 0.71], *p* = 0.7, two RCTs, N = 76) and long-term (8 weeks) antimanic efficacy (SMD = 0.00, 95%CI = [−0.51, 0.51], *p* = 1.00, one RCT, N = 59, Figure 3 and Figure S5).

#### 3.4.2. Tolerability (Discontinuation Due to Adverse Effects)

**Figure 4:**
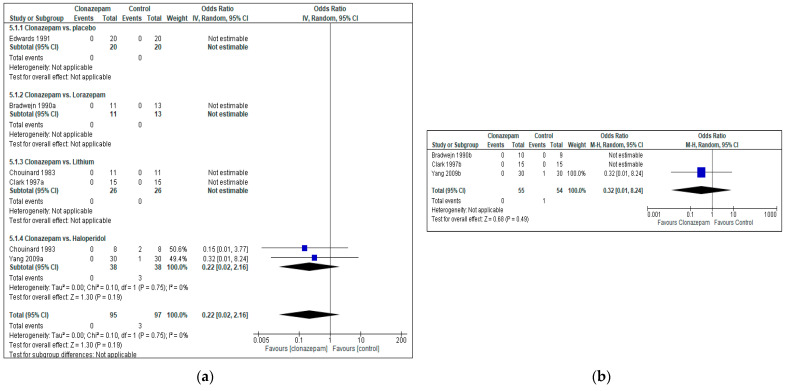
Tolerability (discontinuation due to adverse effects, measured as the proportion of patients who dropped out due to adverse effects): Clonazepam vs. any other pharmacotherapy, including placebo. (**a**): acute effects (up to 3 weeks). Bradwejn 1990a [47], Clark 1997a [49] and Yang 2009a [50] present the acute effects (endpoint up to 3 weeks). (**b**): Bradwejn 1990b [47], Clark 1997b [49] and Yang 2009b [50] present the long-term effects (endpoint more than 3 weeks of continuous treatment).

Clonazepam was well tolerated both acutely and in the long term. Dropouts due to the adverse effects of Clonazepam were not reported in any of the included studies (zero events), whereas four dropouts due to adverse effects were reported in total for any other pharmacotherapy, all among studies using Haloperidol as the comparator (four events). The difference was not statistically significant both in terms of acute effects, as well as in the medium- and the long-term (Acute: OR = 0.22, 95%CI = [0.02, 2.16], *p* = 0.19, six RCTs, N = 192, Figure 4a and Figure S7. Medium and long term: OR = 0.32, 95%CI = [0.01, 8.24], *p* = 0.49, three RCTs, N = 109, Figure 4b and Figure S6).

#### 3.4.3. Acceptability (All Cause Discontinuation)

**Figure 5:**
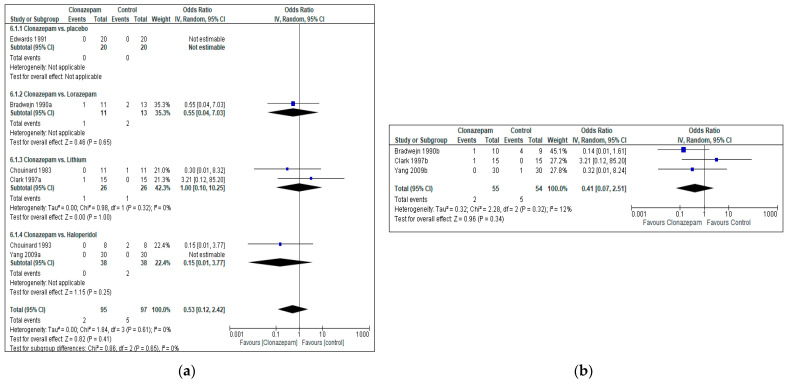
Acceptability (all cause discontinuation, measured as the proportion of patients who dropped out due to any reason): Clonazepam vs. any other pharmacotherapy, including placebo. (**a**): acute effects (up to 3 weeks). Bradwejn 1990a [47], Clark 1997a [49] and Yang 2009a [50] present the acute effects (endpoint up to 3 weeks). (**b**): long-term effects (more than 3 weeks of continuous treatment). Bradwejn 1990b [47], Clark 1997b [49] and Yang 2009b [50] present the medium and long-term effects (endpoint more than 3 weeks of continuous treatment).

Clonazepam was found to be an overall acceptable treatment acutely and in the long term, with a total of 3 dropouts due to any reason among all included studies vs. 10 dropouts due to any reason for any other pharmacotherapy, including placebo. The difference was not statistically significant both acutely and in the medium and long term (Acute: OR = 0.53, 95%CI = [0.12, 2.42], *p* = 0.41, N = 192, Figure 5a and Figure S8. Medium and long term: OR = 0.41, 95%CI = [0.07, 2.51], *p* = 0.34, N = 109, Figure 5b and Figure S7).

### 3.5. Secondary Outcomes

#### 3.5.1. Response to Treatment (Dichotomous)

Only two RCTs reported usable data [47,50] Lorazepam was found to be superior to Clonazepam (OR = 0.14, 95%CI = [0.02, 0.93], *p* = 0.04, one RCT, N = 24, Figure S8), albeit marginally, and there was no difference between Clonazepam and Haloperidol (Figure S9).

#### 3.5.2. Remission (Dichotomous)

Only two RCTs provided data [47,50]. There was a trend for superiority of Lorazepam over Clonazepam acutely and the difference became marginally significant, in favor of Lorazepam, in the medium term (28 days, Figure S10 in Supplementary Materials). There was no difference between Clonazepam and Haloperidol in the long term (16 weeks, Figure S11 in Supplementary Materials).

#### 3.5.3. Efficacy—Global State

Four RCTs provided data [45,46,49,50]. There were no differences between Clonazepam and both Lithium and Haloperidol, acutely, as well as in the long term (Figures S12 and S13 in the Supplementary Materials).

#### 3.5.4. Efficacy—Functioning

Only two RCTs provided data [49,50]. No significant differences between Clonazepam and Lithium or Haloperidol were identified acutely (Clonazepam vs. Lithium) and in the long term (16 weeks, Clonazepam vs. Haloperidol as add-on to Valproate, [50]). The results are presented in Figures S14 and S15 in the Supplementary Materials.

#### 3.5.5. Efficacy—Psychotic Symptoms

Three RCTs provided data [45,46,48]. There were no significant differences between Clonazepam and placebo (N = 40, Figure S16), Lithium (N = 30, Figure S17), and Haloperidol (N = 19, Figure S18).

#### 3.5.6. Total Antipsychotic Dose Utilized and/or Use of As-Required Antipsychotics during the Course of Treatment

Three RCTs provided usable data [45,47,48]. No significant differences in as-required antipsychotic use [45,47] and total antipsychotic dose utilized during the course of treatment [45,48] were identified (Figures S19–S22).

#### 3.5.7. Total Mood Stabilizer Dose Utilized/Plasma Levels in Case of Add-on Treatment

Only one RCT provided usable data [50]. The study compared the combination of Clonazepam and Valproate to the combination of Haloperidol and Valproate. Valproate plasma levels were significantly lower in the Haloperidol group during the acute phase of treatment (endpoint = 14 days, SMD = 0.62 [0.10, 1.14], *p* = 0.02, N = 60, Figure S20), but the difference was no longer significant in the long term (endpoint = 16 weeks, N = 59, Figure S22).

#### 3.5.8. Tolerability—Specific Adverse Effects

The most commonly reported adverse effect of Clonazepam among the included studies was sedation (i.e., sleepiness, somnolence, drowsiness), reported in three RCTs [45,46,47], all examining acute antimanic effects. Clonazepam was significantly more sedative than Lithium (OR = 38.33, 95%CI = [1.79–820.13], *p* = 0.02, N = 11) and marginally more sedative than Lorazepam (OR = 6.6, 95%CI = [0.97–44.93], *p* = 0.05, N = 24), but there were no differences between Clonazepam and Haloperidol (Figure S24).

**Figure 6:**
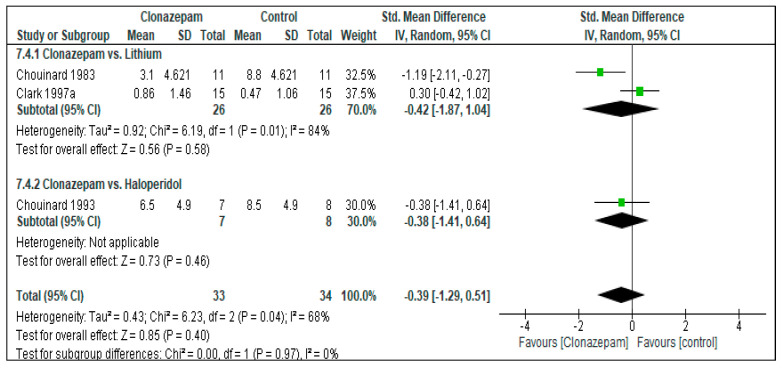
Severity of extrapyramidal adverse effects in the short-term (up to 3 weeks of treatment): Clonazepam vs. any other pharmacotherapy, including placebo. Clark 1997a [49]: acute effects (endpoint up to 3 weeks).

In terms of the occurrence of extrapyramidal adverse effects, three RCTs provided usable data [45,46,49]. There were no differences between Clonazepam and Lithium or Haloperidol (Figure 6 and Figure S25 in the Supplementary Materials). All studies provided data for the short term. No usable long-term data were identified.

Other adverse effects reported among the included RCTs included tremor, ataxia (reported in two RCTs [45,48], the former provided continuous data using a rating scale and the latter the number of patients exhibiting the adverse effect, N = 1 and N = 4, respectively), and blurred vision, reported in one RCT [48].

#### 3.5.9. Other Secondary Outcomes Mentioned in Our a Priori Written Protocol

No data were available for the outcomes of manic relapse in the course of follow-up, suicidality, quality of life, and insomnia.

### 3.6. Publication Bias

When fewer than ten studies are included in a meta-analysis, asymmetry testing for funnel plots should not be employed [51]. Since only six studies were included in this work, we could not assess publication bias using funnel plots.

### 3.7. Subgroup and Sensitivity Analyses for the Primary Outcomes

Applying a fixed effects model did not affect the results of the primary outcomes (Figures S26–S31). The limited number of included studies, often resulting in a single study per comparison, did not allow us to perform the remaining a priori planned subgroup and sensitivity analyses.

## 4. Discussion

To the best of our knowledge, the present study is the only comprehensive systematic review and meta-analysis examining the antimanic efficacy, tolerability, and acceptability of Clonazepam in the acute phase of treatment, as well as in the medium and long term.

We found that Clonazepam may be superior to placebo and equally efficacious with both Lithium and Haloperidol, both in the acute phase of treatment (up to 3 weeks) and in the medium and long term (longer than 3 weeks). Additionally, Clonazepam may be considered an acceptable and well-tolerated treatment. However, it should be noted that all our comparisons were underpowered, and the majority of outcomes had only one study per comparison with minimal sample sizes. This limits the generalizability of our findings and prevents us from drawing safe clinical conclusions.

### 4.1. Scarcity of Data

Perhaps the most notable finding is that, despite conducting a comprehensive search and making efforts to include non-randomized controlled studies and the gray literature, we were only able to identify six controlled studies that compared Clonazepam with either a placebo or another pharmacotherapy. These studies were all RCTs and included a total of 192 patients. It is known that effect sizes are markedly changeable when less than 1000 participants are included in a psychopharmacotherapy meta-analysis, and they stabilize when this threshold is reached [52]. None of our comparisons reached this threshold, which is a considerable limitation.

In comparison to the sole existing meta-analysis [30], which was published approximately 20 years ago, we only discovered two additional studies. This scarcity of research could potentially be attributed to the lack of endorsement for the use of benzodiazepines in the current guidelines, which emphasize the risks of dependency and cognitive impairment [1,17]. Consequently, there may be reduced investment in conducting clinical trials involving benzodiazepines. Another concern that should be considered is that of publication bias. Studies supporting the use of benzodiazepines might be less likely to be published in light of the existing guidance. The above limitations may lead to an incomplete picture of the evidence that guides clinical decision making.

Our findings indicate that when it comes to the prescription of Clonazepam, clinical decision making relies on empirical evidence and clinical experience. We believe that this is particularly noteworthy, given the widespread use of Clonazepam for the treatment of acute mania [20]. It underscores the existing schism between the research and production of an evidence base and the reality of clinical practice.

### 4.2. Other Limitations

Additional limitations to consider when evaluating the aforementioned efficacy findings include (i) the relatively short duration of most included trials; (ii) the potential influence of older studies and studies conducted in China on effect sizes; (iii) the fact that most studies allowed the use of antipsychotics on an “as-required” basis, even when examining interventions as monotherapies; and (iv) the utilization of relatively high doses of Clonazepam compared to current standards.

#### 4.2.1. Duration of the Included Trials

The duration of the included studies may hold particular significance in explaining our findings of no difference in efficacy between Clonazepam and Lithium. The onset of action of Lithium is relatively slow. Dose adjustments during the initial weeks of treatment until reaching the therapeutic range are common [53]. On the other hand, Clonazepam exhibits an acute onset of action [23]. This difference may have influenced the effect sizes in the trial conducted by Chouinard and colleagues [45], which only lasted 10 days for each intervention. However, the trial conducted by Clark and colleagues [49], which also compared Lithium with Clonazepam, had a relatively longer duration of 28 days, and the efficacy of Clonazepam did not differ from the efficacy of Lithium at day 28.

#### 4.2.2. The Potential Effect of Old Studies and Studies from Mainland China

It is known that older trials might show exaggerated results due to suboptimal randomization or blinding quality and publication bias [54]. It has also been reported that studies from mainland China may frequently employ randomization methods that differ from the internationally approved ones. Furthermore, the methods are often not adequately described [55,56,57]. All but one study included in this review were old (published between 1983 and 1993), and the only more recent study was a study from China. Indeed, our risk of bias assessment indicated that no study was deemed to have an overall low risk of bias, and the details of randomization and allocation concealment were unclear in all the included studies. This may have affected the reported effect sizes.

#### 4.2.3. Concurrent Antipsychotic Prescribing in Trials Examining Clonazepam as a Monotherapy

It should be noted that all but two studies examining Clonazepam vs. other pharmacotherapy allowed the use of as-required antipsychotic treatment [45,48,49,50]. This could potentially confound the results and make it challenging to isolate the specific effects of Clonazepam. Furthermore, one of the two studies that did not permit a concurrent as-required antipsychotic [46] reports that antipsychotics had been administered to 75% of the patients on the day of the trial. This is significant because the duration of the trial was only one day, and it examined the antimanic efficacy of rapid tranquilization using Clonazepam vs. Haloperidol. The above studies showed the superiority of Clonazepam over placebo and no difference compared to Lithium and Haloperidol.

In the only study that did not permit as-required antipsychotic prescribing for the duration of the trial of Lorazepam vs. Clonazepam monotherapy (up to day 14) [47], there was a trend for the superiority of Lorazepam over Clonazepam in our continuous efficacy outcomes and a statistically significant difference in favor of Lorazepam in our dichotomous outcomes of response to treatment and remission.

This may indicate that Clonazepam is more efficacious as an adjunct to antipsychotics compared to monotherapy, and further research is required to test this hypothesis.

#### 4.2.4. Dose of Clonazepam Utilized

The dose of Clonazepam used in some of the included studies was very high by today’s standards. When Clonazepam was compared to Lithium as a monotherapy, a range between 8 mg and 16 mg daily was used in the study by Clark and colleagues [49], and an average daily dose of 10.4 ± 6.7 mg was used in the study by Chouinard and colleagues [45]. An amount of 10 mg of Clonazepam is considered equivalent to 50–200 mg of Diazepam [58]. The maximum usually prescribed daily dose of Clonazepam is 8 mg according to the British National Formulary [59].

In that regard, it is also important to consider that in the trial of Bardwejn and colleagues [47], the mean Clonazepam daily dose was 14.2 ± 6.7 mg and the mean Lorazepam daily dose was 13 ± 5.1 mg at day 14 of monotherapy with each agent. The average Lorazepam dose was therefore equivalent to 65–130 mg of Diazepam [58].

In contrast to the aforementioned studies, a fixed daily dose of 6 mg of Clonazepam monotherapy was sufficient to achieve antimanic efficacy within 5 days in the trial conducted by Edwards and colleagues [48], which compared the drug to a placebo. When Clonazepam was compared to Haloperidol as an adjunctive treatment to Valproate, Clonazepam doses ranging from 2 to 6 mg daily were found to be effective in achieving antimanic efficacy. Lastly, in the only study that used intramuscular Clonazepam versus intramuscular Haloperidol for rapid tranquilization [46], a mean of 5.4 ± 1.2 mg of Clonazepam was administered, but, as mentioned above, the patients included in this study were on regular antipsychotic treatment.

The above findings indicate that high doses of Clonazepam may be necessary for it to exert its antimanic efficacy when prescribed as a monotherapy, whereas lower doses, in line with current standards, may be sufficient when it is used as an augmentation to Valproate. However, it is important to emphasize once again the limited amount of data available. More and larger-scale studies are required to draw safe and clinically applicable conclusions.

#### 4.2.5. Discussion of the Outcomes of Tolerability and Acceptability, and Implications for Antimanic Mechanism of Action

A noteworthy finding is that, despite the high doses used in the included studies and the polytherapy utilized in most cases, Clonazepam was well tolerated and considered an acceptable treatment, both acutely and in the medium to long term. However, the availability of long-term data was minimal.

None of the included studies reported dropouts due to the adverse effects of Clonazepam. Although it was significantly more sedative compared to Lithium and marginally more sedative compared to Lorazepam, it did not exhibit greater sedation compared to Haloperidol, despite the utilization of very high doses. This may suggest that sedation is not the sole mechanism through which Clonazepam exerts its antimanic effect. Indeed, it has been postulated that Clonazepam may inhibit dopamine release [25] and may suppress d-amphetamine-induced manic behavior [24]. Our findings suggest that Clonazepam may be equally efficacious with Haloperidol in treating psychotic symptoms (Figure S19), indicating its potential antipsychotic properties. Interestingly, no significant differences in efficacy for treating psychotic symptoms were observed when comparing Clonazepam to a placebo and Lithium (Figures S17 and S18). However, it should be noted that these results are confounded by the use of as-required antipsychotic treatment in both arms of these trials [48,49]. As a result, a definitive conclusion cannot be drawn, but further research in this area would be valuable.

It has been argued that one of the benefits of treating acute mania with Clonazepam is that it reduces the required dose of antipsychotics, thereby mitigating the risk of EPSEs [26]. It is true that Clonazepam has been successfully used to treat EPSEs, including tardive dyskinesia [60] and akathisia [61]. Our findings indicate that Clonazepam was not superior to other pharmacotherapy (including Lithium and Haloperidol, Figure 6 and Figure S26) in terms of the severity of EPSEs. This may be because we found no significant differences between Clonazepam and other pharmacotherapy in terms of the total antipsychotic dose utilized (Figures S20 and S21) and the proportion of patients requiring antipsychotic treatment when Clonazepam is compared to other pharmacotherapy (Figures S22 and S23). However, it is important to mention that, due to scarcity of data, minimal sample sizes were included in each of these comparisons, which may be the reason why statistical significance was not reached. The raw data appear more impressive. Chouinard and colleagues [45] reported a mean total daily dose of 55 mg of Haloperidol in the Clonazepam group vs. 165 mg in the Lithium group, with 4 out of 11 vs. 7 out of 11 participants requiring rescue antipsychotic treatment over 10 days of treatment. They reported the difference in mean doses as statistically significant.

### 4.3. Clinical Implications

Based on the above considerable limitations, we can neither strongly recommend nor refute the clinical utility of Clonazepam for the treatment of acute mania in daily clinical practice. The existing evidence, however, indicates that Clonazepam may serve as a well-tolerated and acceptable treatment when used as an augmentation to antipsychotics or mood stabilizers, particularly in cases where other options are not acceptable, tolerable, or contraindicated, or when significant risks to the patient’s physical health are involved. Given its potential for rapid antimanic efficacy, it may also be a valuable augmentation strategy for mood stabilizers during the acute phase.

In addition, we suggest that guidelines, local treatment policies, and protocols might consider adopting a more balanced approach. This entails acknowledging the scarcity of evidence while recognizing the widespread empirical use of Clonazepam in clinical practice. By doing so, they can acknowledge both the potential efficacy and associated risks of Clonazepam and benzodiazepines in general, rather than solely focusing on the latter aspect. This approach may better reflect the realities of real-world clinical practice.

## 5. Conclusions

The existing RCT evidence suggests that Clonazepam may be an efficacious, acceptable, and well-tolerated treatment for acute mania. However, there are considerable limitations that compromise the generalizability and clinical utility of these findings. The data are limited, resulting in a single study per comparison for many outcomes. Furthermore, the existing studies are old and the details of randomization and allocation concealment were unclear in all the identified studies, with no study evaluated as having a low risk of bias. The reported doses required to achieve antimanic efficacy when Clonazepam is prescribed as a monotherapy are considered very high by today’s standards. Lastly, the majority of studies allowed concomitant use of antipsychotic medication, which may confound the results and make it challenging to isolate the specific effects of Clonazepam.

In light of the above limitations, we are unable to firmly endorse or dismiss the clinical effectiveness of Clonazepam for treating acute mania. We however suggest that it may be a viable add-on treatment with antipsychotics or mood stabilizers, especially when other options are unsuitable or unsafe.

Considering the current prevalence of benzodiazepine use for the treatment of mania, especially in inpatient settings, we strongly advocate for more and larger-scale studies to investigate the acute and also the long-term antimanic efficacy, acceptability, and tolerability of benzodiazepines in general, and Clonazepam in particular.

## Figures and Tables

**Table 1:** Characteristics of included studies.

First Author and Year of Publication	Study Location	Design	Duration	Diagnostic Criteria	Baseline Severity of Illness	Information about Participants’ Age (Years)	Setting	Arms and Number of Participants per Arm	Information about Clonazepam Preparation and Dose
Edwards 1991 [48]	New Zealand	Double-blind, parallel RCT	5 days	DSM-III	Mean CGR-mania = 4.75 (1.19)	Mean age per group *: 34.2 and 31.7	Inpatient	Clonazepam (N = 20) vs. placebo (N = 20), monotherapy, as required antipsychotic allowed.	Oral, 6 mg daily, fixed dose
Bradwejn 1990 [47]	Canada	Double-blind, parallel RCT	1st Phase: 14 days. 2nd Phase: 28 days	DSM-III	Mean CGI per group: 5 and 5.2 *	Mean age/SD: 41.68 (11.93)	Inpatient	1st Phase: Clonazepam (N = 11) vs. Lorazepam (N = 13), monotherapy 2nd Phase: Clonazepam + Lithium vs. Lorazepam + Lithium, allowed as required antipsychotic	Oral, mean = 14.2 (6.7) mg daily (range: 6–20 mg daily)
Chouinard 1983 [45]	Canada	Double-blind, cross-over RCT	10 days	Spitzer 1978	Mean CGI-mania = 8.2 (0.6)	Median = 43 *	Inpatient	Clonazepam vs. Lithium (N = 12), monotherapy, but as required antipsychotic allowed	Oral, mean = 10.4 (6.7) mg daily (range 4–16 mg daily)
Clark 1997 [49]	South Africa	Open (unblinding due to design limitations) parallel RCT	28 days	DSM-IV	Mean CGI = 4.63 (0.624)	18–65 *	Inpatient	Clonazepam (N = 15) vs. Lithium (N = 15), monotherapy, but as required antipsychotics allowed.	Oral, range: 8–16 mg *
Chouinard 1993 [46]	Canada	Double-blind parallel RCT	1 day (2 h)	DSM-III	Mean CGI = 5.45 (1.15)	34.95 (10.01)	Inpatient	Clonazepam (N = 8) vs. Haloperidol (N = 8), 75% of patients on regular antipsychotics	Intramuscular injection, mean = 5.4 (1.2)mg
Yang 2009 [50]	China	Single-blind, parallel RCT	16 weeks	DSM-IV	Mean CGI = 5.25 (0.45)	Mean = 31.85 (10.27)	Inpatient	Clonazepam + Valproate (N = 30) vs. Haloperidol + Valproate (*n* = 30), as required antipsychotics not allowed, but as required benzodiazepines and hypnotics allowed.	Oral, range: 2–6 mg daily *

RCT: randomized controlled trial. N: number of participants. CGI: Clinical Global Impression scale. CGR: Clinician’ Global Rating scale. Mg: milligrams. * no further information available to allow imputation.

## Data Availability

Not applicable.

## Supplementary Materials

The following supporting information can be downloaded at: https://www.mdpi.com/article/10.3390/jcm12185801/s1, Supplement A: Protocol of the systematic review and meta-analysis; Supplement B: Search Strategy (databases); Supplement C: PRISMA checklist; Supplement D: Figure S1: PRISMA flow diagram; Supplement E: Table S1: List of excluded controlled studies and studies awaiting assessment; Supplement F: Forest plots for all outcomes; Figure S2: Response to treatment, continuous—Clonazepam vs. Placebo; Figure S3: Response to treatment, continuous—Clonazepam vs. Lorazepam; Figure S4: Response to treatment, continuous—Clonazepam vs. Lithium; Figure S5: Response to treatment, continuous—Clonazepam vs. Haloperidol; Figure S6: Tolerability; Figure S7: Acceptability; Figure S8: Response to treatment, dichotomous—Clonazepam vs. Lorazepam; Figure S9: Response to treatment, dichotomous—Clonazepam vs. Haloperidol; Figure S10: Remission, dichotomous—Clonazepam vs. Lorazepam; Figure S11: Remission, dichotomous—Clonazepam vs. Haloperidol; Figure S12: Efficacy, global state—Clonazepam vs. Lithium; Figure S13: Efficacy, global state—Clonazepam vs. Haloperidol; Figure S14: Functioning—Clonazepam vs. Lithium; Figure S15: Functioning—Clonazepam vs. Haloperidol; Figure S16: Psychotic symptoms—Clonazepam vs. Placebo; Figure S17: Psychotic symptoms—Clonazepam vs. Lithium; Figure S18: Psychotic symptoms—Clonazepam vs. Haloperidol; Figure S19: Total antipsychotic dose—Clonazepam vs. Placebo; Figure S20: Total antipsychotic dose—Clonazepam vs. Lithium; Figure S21: As required antipsychotic use—Clonazepam vs. Lithium; Figure S22: As required antipsychotic use—Clonazepam vs. Lorazepam; Figure S23: Valproate plasma levels—Clonazepam vs. Haloperidol; Figure S24: Sedation—Clonazepam vs. any other pharmacotherapy; Figure S25: Extrapyramidal—Clonazepam vs. any other pharmacotherapy; Supplement G: Sensitivity analysis for the primary outcomes: fixed effects model; Figure S26: Response to treatment, continuous—Clonazepam vs. Placebo; Figure S27: Response to treatment, continuous—Clonazepam vs. Lorazepam; Figure S28: Response to treatment, continuous—Clonazepam vs. Lithium; Figure S29: Response to treatment, continuous—Clonazepam vs. Haloperidol; Figure S30: Tolerability; Figure S31: Acceptability.
