# Supplementary material for: Antimanic Efficacy, Tolerability, and Acceptability of Clonazepam: A Systematic Review and Meta-Analysis
Source: J Clin Med. 2023 Sep 6;12(18):5801. doi: 10.3390/jcm12185801 (PMC10531794; doi:10.3390/jcm12185801)
Supplement: Supplementary file 1 [file jcm-12-05801-s001.zip › jcm-2534372-supplementary.pdf]

Supplementary material

Table of contents

Title of supplement		Page Number
A.	Protocol of the systematic review and meta-analysis	2
B.	Search Strategy (databases)	7
C.	PRISMA checklist	12
D.	Figure S1: PRISMA flow diagram	14
E.	Table S1: List of excluded controlled studies and studies awaiting assessment	15
F.	Forest Plots for all outcomes	16
	Figure S2: Response to treatment, continuous – Clonazepam vs. Placebo	16
	Figure S3: Response to treatment, continuous – Clonazepam vs. Lorazepam	16
	Figure S4: Response to treatment, continuous – Clonazepam vs. Lithium	17
	Figure S5: Response to treatment, continuous – Clonazepam vs. Haloperidol	17
	Figure S6: Tolerability	18
	Figure S7: Acceptability	19
	Figure S8: Response to treatment, dichotomous: Clonazepam vs. Lorazepam	20
	Figure S9: Response to treatment, dichotomous: Clonazepam vs. Haloperidol	20
	Figure S10: Remission, dichotomous: Clonazepam vs. Lorazepam	20
	Figure S11: Remission, dichotomous: Clonazepam vs. Haloperidol	21
	Figure S12: Efficacy, global state: Clonazepam vs. Lithium	21
	Figure S13: Efficacy, global state: Clonazepam vs. Haloperidol	21
	Figure S14: Functioning: Clonazepam vs. Lithium	22
	Figure S15: Functioning: Clonazepam vs. Haloperidol	23
	Figure S16: Psychotic symptoms: Clonazepam vs. Placebo	23
	Figure S17: Psychotic symptoms: Clonazepam vs. Lithium	23
	Figure S18: Psychotic symptoms: Clonazepam vs. Haloperidol	23
	Figure S19: Total antipsychotic dose: Clonazepam vs. Placebo	24
	Figure S20: Total antipsychotic dose: Clonazepam vs. Lithium	24
	Figure S21: As required antipsychotic use: Clonazepam vs. Lithium	24
	Figure S22: As required antipsychotic use: Clonazepam vs. Lorazepam	24
	Figure S23: Valproate plasma levels: Clonazepam vs. Haloperidol	25
	Figure S24: Sedation: Clonazepam vs. any other pharmacotherapy	26
	Figure S25: Extrapyramidal: Clonazepam vs. any other pharmacotherapy	26
G.	Sensitivity Analysis for the primary outcomes: Fixed effects model	26
	Figure S26: Response to treatment, continuous – Clonazepam vs. Placebo	26
	Figure S27: Response to treatment, continuous – Clonazepam vs. Lorazepam	26
	Figure S28: Response to treatment, continuous – Clonazepam vs. Lithium	38
	Figure S29: Response to treatment, continuous – Clonazepam vs. Haloperidol	28
	Figure S30: Tolerability	29
	Figure S31: Acceptability	30

A: Protocol of the systematic review and meta-analysis

Antimanic efficacy, tolerability and acceptability of clonazepam: a systematic review and meta-analysis

Andreas Lappas, Bartosz Helfer, Katarzyna Henke-Ciążyńska, Myrto Samara, Nikos Christodoulou. Antimanic efficacy, tolerability and acceptability of clonazepam: a systematic review and meta-analysis. PROSPERO 2023 CRD42023432231 Available from: https://www.crd.york.ac.uk/prospero/display_record.php?ID=CRD42023432231

Review question

The objective of this systematic review and meta-analysis is to examine the antimanic efficacy, tolerability and acceptability of Clonazepam in comparison with placebo or other antimanic pharmacological treatments.

Searches

The following sources will be searched without any restrictions in terms of language or publication period and status:

1. Electronic databases: multiple systematic searches will be conducted using MEDLINE (via Ovid), Embase, APA PsycINFO, Cochrane Central Register of Controlled Trials (CENTRAL), ClinicalTrials.gov and WHO International Clinical Trials Registry Platform (ICTRP).

2. Previous reviews: we will search for previously published reviews for relevant studies.

3. Reference searching: we will manually review the references of all identified studies.

Types of study to be included

We will include studies that have compared clonazepam to a control pharmacotherapy condition, such as a different antimanic pharmacological treatment or a placebo. Eligible study designs will include individual, and cluster randomized controlled trials (RCTs), quasi-randomized trials, and non-randomized controlled trials. We will include all relevant studies that meet our broad inclusion criteria, irrespective of the use of blinding (open-label, single-, or double-blind) and the reporting of usable data or not. We will include both published and unpublished studies. There will be no language restriction to mitigate the risk of language bias and maximize the amount of usable data.

Condition or domain being studied

Operationalized or clinical diagnosis of Bipolar Affective Disorder, Schizoaffective disorder any other form of affective psychosis with mania (any form of ICD, DSM, Feighner 1972, Spitzer 1978, or clinical diagnosis as reported by the authors). We will exclude studies that did not use operationalized criteria in a sensitivity analysis.

Participants with the following subtypes of bipolar disorder will be included:

1. Manic episode with or without psychotic symptoms, including treatment resistant mania (however this is defined)

2. Mixed affective state

3. Rapid cycling bipolar disorder

We will also include patients with mania in the context of puerperal psychosis, schizoaffective disorder, or any other form or affective psychosis. We will not exclude organic mania (in the context of delirium or of another aetiology, for example steroid-induced mania, thyrotoxicosis etc.).

A subgroup analysis will be attempted based on primary diagnosis, depending on availability of data.

Participants/population

Operationalized or clinical diagnosis of Bipolar Affective Disorder, Schizoaffective disorder any other form of affective psychosis with mania (any form of ICD, DSM, Feighner 1972, Spitzer 1978, or clinical diagnosis as reported by the authors). We will exclude studies that did not use operationalized criteria in a sensitivity analysis.

Participants with the following subtypes of bipolar disorder will be included:

1. Manic episode with or without psychotic symptoms, including treatment resistant mania (however this is defined)

2. Mixed affective state

3. Rapid cycling bipolar disorder

We will also include patients with mania in the context of puerperal psychosis, schizoaffective disorder, or any other form or affective psychosis. We will not exclude organic mania (in the context of delirium or of another aetiology, for example steroid-induced mania, thyrotoxicosis etc.).

A subgroup analysis will be attempted based on primary diagnosis, depending on availability of data.

There will be no restrictions in terms of gender, ethnicity, age, or setting.

Intervention(s), exposure(s)

Clonazepam, used in any form or preparation (for example, oral, intramuscular), either as monotherapy, or as augmentation of other pharmacotherapy.

Comparator(s)/control

Placebo or any other antimanic/tranquilizing drug, such as lithium, antipsychotics, other benzodiazepines.

Main outcome(s)

1. Efficacy - response to treatment (continuous), measured as mean change scores (will be given preference) or endpoint scores (if data on change scores not available) in symptoms of mania measured by YMRS (will be given preference) or any other similar validated rating scale, or authors' definitions if data are not available
2. Tolerability (dichotomous): number of dropouts due to treatment emergent adverse effects (between the first treatment dose and endpoint)

3. Acceptability (dichotomous): number of dropouts due to any reason (all-cause discontinuation)

Measures of effect
We will aim to divide all outcomes into acute (up to 3 weeks), and long term (over 3 weeks), with acute being our primary outcomes. For dichotomous outcomes we will calculate a standard estimation of the risk ratio (RR) and its 95% confidence interval (CI). For continuous outcomes we will estimate the mean difference (MD) between groups. We prefer not to calculate effect size measures such as standardized mean difference (SMD). However, if scales of significant similarity are used, we will presume there is a small difference in measurement, and we will calculate SMDs.

Additional outcome(s)

1. Efficacy (dichotomous): response to treatment, defined as a reduction of ≥50% in mean YMRS (Young Mania Rating Scale, will be given preference) or any other similar validated rating scale, compared to baseline, or however defined by the authors if data are not available
2. Remission (dichotomous), proportion of patients in remission following treatment. Remission will be defined as YMRS score ≤12 (will be given priority), or equivalent on other validated scale, or however defined by the authors if data are not available

3. Tolerability – specific adverse effects (dichotomous): proportion of participants experiencing specific treatment emergent adverse effects

4. Relapse (dichotomous): proportion of participants experiencing relapse, however defined by the authors

5. Global state (continuous): mean change scores (will be given preference) or endpoint scores in symptoms of mania measured by the CGIs (Clinical Global Impressions) scale (will be given preference) or any other similar validated rating scale

6. Use of rescue medication for aggressive behaviour (dichotomous)

7. Suicidality (dichotomous or continuous)

8. Overall and social functioning (continuous): change score (preferable) or endpoint scores, measured by rating scales such as the Global Assessment of Functioning or the Psychosocial Performance Scale, or any other published rating scale.

9. Quality of life (continuous). Any published rating scale (e.g. “Heinrichs quality of life scale”, Quality of Life Scale).

10. Insomnia (continuous): change (preferable) or endpoint values of total nocturnal sleep time, defined as the total amount of time of sleep as perceived by participants

11. Any other outcome

Measures of effect
We will aim to divide all outcomes into acute (up to 3 weeks), and long term (over 3 weeks), with acute being our primary outcomes. For dichotomous outcomes we will calculate a standard estimation of the risk ratio (RR) and its 95% confidence interval (CI). For continuous outcomes we will estimate the mean difference (MD) between groups. We prefer not to calculate effect size measures such as standardized mean difference (SMD). However, if scales of significant similarity are used, we will presume there is a small difference in measurement, and we will calculate SMDs.

Data extraction (selection and coding)

Review of eligibility of studies: at least two reviewers will independently inspect all abstracts and then the full texts identified from the searches. The process will be

facilitated using Rayyan (https://www.rayyan.ai/). Conflicts will be resolved by discussions between the reviewers. At least two reviewers will independently decide whether the studies meet the inclusion criteria.

Data extraction: At least two reviewers will independently extract data from all selected trials, using a specifically designed data entry form. Conflicts between the reviewers will be resolved by discussion. All decisions will be documented.

We will attempt to contact authors through an open-ended request to obtain missing information or for clarification. In the case of crossover trials, we will use the first crossover phase to avoid the problem of carryover effects, if possible. Otherwise, we will include the results as presented by the authors, but only if there is an adequate washout period between the different phases, defined as a minimum of 5 times the elimination half-life of each drug.

Risk of bias (quality) assessment

At least two review authors will work independently to assess the risk of bias. The Cochrane Risk of Bias Tool for Randomized Trials will be used for RCTs and the Risk Of Bias In Non- randomized Studies (ROBINS-I) tool will be used for non-randomized studies.

Strategy for data synthesis

We will employ a random-effects model for the analyses (Der-Simonian 1986). We understand that there is no closed argument for preference of fixed or random effects model. The random-effects model is usually more conservative in terms of statistical significance, although as a disadvantage it puts added weight onto smaller studies which can either inflate or deflate the effect size. Therefore, we will examine in a sensitivity analysis whether using a fixed model markedly changes the results of the primary outcomes. All analyses will be on an intention-to-treat (ITT) basis. We will only use completed analyses if ITT data are unavailable. We will address this issue in the Risk of bias in included studies tool. We will also conduct a sensitivity analysis by excluding studies which presented only completer data.
The effect size for dichotomous outcomes will be assessed by calculating the Risk Ratio (RR). The effect size for continuous outcomes will be assessed by calculating the weighted mean difference (MD). If data are presented in different scales, then the standardized mean difference (SMD) will be calculated instead. Missing standard deviations will be calculated from standard errors or estimated from confidence intervals, t-values, or p-values as described in Section 7.7.3 of the Cochrane Handbook for Systematic Reviews. Heterogeneity: All included trials will be considered initially. Heterogeneity will be investigated by inspection of the forest plots. Statistical heterogeneity will be tested with the χ^2 test and quantified by the I^2 statistics. Potential reasons for heterogeneity will be explored with subgroup analyses, but these will only be conducted on the primary outcomes.

Analysis of subgroups or subsets

Subgroup analysis will be conducted for the primary outcomes only and depending on availability of data.
1. Per primary diagnosis or, if limited availability of data, mania with psychosis vs. mania without psychosis

2. Rapid cycling mania vs. not

3. Treatment resistant mania vs. not

4. Monotherapy vs. add-on drug treatment

5. Children and adolescent participants vs. adults

6. Adult participants older than 65 vs. not

7. Comorbid substance misuse vs. not

8. Any other required in the process of investigating heterogeneity

Sensitivity analyses: for the primary outcomes only and depending on availability of data.

1. Exclusion of studies that did not use operationalised criteria for diagnosis

2. Exclusion of non-randomised studies

3. Fixed effects instead of random effects model

4. Exclusion of studies with imputed data

5. Exclusion of sponsored studies

Contact details for further information

Dr Andreas Lappas
andreaslappaswork@gmail.com

B. Search Strategy

Database: Ovid MEDLINE(R) ALL <1946 to April 27, 2023> Search Strategy:

1 clonazepam.mp. (4911)
2 (Klonopin or Rivotril or Paxam or Rivatril or Ravotril or Clonex or Clonotril or Kenoket or Kriadex or Lonazep or Antelepsin or Iktorivil or Linotril or Clonam).tw,kf. (73)
3 or/1-2 (4923)
4 bipolar.mp. (90136)
5 ("bipolar affective disorder" or "mania" or "manic" or "manic depression" or "cyclothym*" or "rapid cycling bipolar" or "manic depression" or "hypomania" or "affective psychosis" or "circular insanity" or "hyperthymia").tw,kf. (24680)
6 ("acute mania" or "acute manic").tw,kf. (1278)
7 (chronic adj5 (bipolar or mania or manic)).tw,kf. (834)
8 (relapse adj5 (bipolar or mani*)).tw,kf. (879)
9 exp Affective Disorders, Psychotic/ (2316)
10 (schizo* adj5 (mania or manic or mixed)).tw,kf. (2090)
11 ("mixed mood episode" or "mixed affective state" or "dysphoric mania" or "mixed state psychosis").tw,kf. (87)
12 mania.mp. (11951)
13 manic.mp. (12396)
14 ((puerperal or "postpartum" or "post-partum" or perinatal or "post-natal" or obstetric) adj5 (mania or manic or mixed or psycho*)).tw,kf. (3121)
15 Ultradian cycling.tw,kf. (30)
16 (agitation adj10 (bipolar or mania or manic)).tw,kf. (282)
17 ("psychomotor hyperactivity" adj10 (bipolar or mania or manic)).tw,kf. (2)
18 ("mani* with psychos*" or "mani* without psychos*").tw,kf. (26)
19 or/4-18 (100645)
20 3 and 19 (173)

Database: Embase <1974 to 2023 April 28> Search Strategy:

1 clonazepam.mp. or exp clonazepam/ (30221)
2 (Klonopin or Rivotril or Paxam or Rivatril or Ravotril or Clonex or Clonotril or Kenoket or Kriadex or Lonazep or Antelepsin or Iktorivil or Linotril or Clonam).mp. (2156)
3 or/1-2 (30237)
4 (rat or rats or mouse or mice or swine or porcine or murine or sheep or lambs or pigs or piglets or rabbit or rabbits or cat or cats or dog or dogs or cattle or bovine or monkey or monkeys or trout or marmoset$1).ti. and animal experiment/ (1222350)
5 (Systematic review not (trial or study)).ti. (257190)
6 (Meta-analysis not (trial or study)).ti. (211464)
7 (review.ab. and review.pt.) not trial.ti. (1108306)
8 we searched.ab. and (review.ti. or review.pt.) (49292)
9 update review.ab. (138)
10 (databases adj4 searched).ab. (61592)
11 or/4-10 (2598863)
12 3 not 11 (27724)
13 bipolar disorder.mp. or exp bipolar disorder/ (85176)
14 affective psychosis.mp. or exp affective psychosis/ (2979)
15 organic psychosis.mp. or exp organic brain syndrome/ (3086)
16 schizoaffective disorder.mp. or exp schizoaffective psychosis/ (14239)
17 puerperal psychosis.mp. or exp puerperal psychosis/ (1436)
18 mania.mp. or exp mania/ or exp bipolar mania/ (92521)
19 ("bipolar affective disorder" or mania or manic or "manic depression" or "cyclothym*" or "rapid cycling bipolar" or "manic depression" or "hypomania" or "affective psychosis" or "circular insanity" or "hyperthymia").ti,ab,tw. (33323)
20 ("acute mania" or "acute manic").ti,ab,tw. (1799)
21 (chronic adj5 (bipolar or mania or manic)).ti,ab,tw. (1329)
22 (relapse adj5 (bipolar or mani*)).ti,ab,tw. (1353)
23 (schizo* adj5 (mania or manic or mixed)).ti,ab,tw. (2808)
24 ("mixed mood episode" or "mixed affective state" or "dysphoric mania" or "mixed state psychosis").ti,ab,tw. (118)
25 ((puerperal or "postpartum" or "post-partum" or perinatal or "post-natal" or obstetric) adj5 (mania or manic or mixed or psycho*)).ti,ab,tw. (4076)
26 Ultradian cycling.ti,ab,tw. (42)
27 (agitation adj10 (bipolar or mania or manic)).ti,ab,tw. (478)
28 ("psychomotor hyperactivity" adj10 (bipolar or mania or manic)).ti,ab,tw. (3)
29 ("mani* with psychos*" or "mani* without psychos*").ti,ab,tw. (49)
30 or/13-29 (122342)
31 12 and 30 (1818)

Database: APA PsycInfo <1806 to April Week 4 2023> Search Strategy:

1 clonazepam.mp. or exp clonazepam/ (1482)

2 (Klonopin or Rivotril or Paxam or Rivatril or Ravotril or Clonex or Clonotril or Kenoket or Kriadex or Lonazep or Antelepsin or Iktorivil or Linotril or Clonam).mp. (14)

3 or/1-2 (1488)

4 (rat or rats or mouse or mice or swine or porcine or murine or sheep or lambs or pigs or piglets or rabbit or rabbits or cat or cats or dog or dogs or cattle or bovine or monkey or monkeys or trout or marmoset$1).ti. and animal experiment/ (39)

5 (Systematic review not (trial or study)).ti. (31457)

6 (Meta-analysis not (trial or study)).ti. (23727)

7 (review.ab. and review.pt.) not trial.ti. (0)

8 we searched.ab. and (review.ti. or review.pt.) (3118)

9 update review.ab. (20)

10 (databases adj4 searched).ab. (7430)

11 or/4-10 (50184)

12 3 not 11 (1476)

13 bipolar disorder.mp. or exp bipolar disorder/ (46818)

14 affective psychosis.mp. or exp affective psychosis/ (1432)

15 organic psychosis.mp. or exp organic brain syndrome/ (92461)

16 schizoaffective disorder.mp. or exp schizoaffective psychosis/ (6279)

17 puerperal psychosis.mp. or exp puerperal psychosis/ (505)

18 mania.mp. or exp mania/ or exp bipolar mania/ (18027)

19 ("bipolar affective disorder" or mania or manic or "manic depression" or "cyclothym*" or "rapid cycling bipolar" or "manic depression" or "hypomania" or "affective psychosis" or "circular insanity" or "hyperthymia").ti,ab,tw. (26169)

20 ("acute mania" or "acute manic").ti,ab,tw. (1203)

21 (chronic adj5 (bipolar or mania or manic)).ti,ab,tw. (743)

22 (relapse adj5 (bipolar or mani*)).ti,ab,tw. (464)

23 (schizo* adj5 (mania or manic or mixed)).ti,ab,tw. (3237)

24 ("mixed mood episode" or "mixed affective state" or "dysphoric mania" or "mixed state psychosis").ti,ab,tw. (86)

25 ((puerperal or "postpartum" or "post-partum" or perinatal or "post-natal" or obstetric) adj5 (mania or manic or mixed or psycho*)).ti,ab,tw. (2454)

26 Ultradian cycling.ti,ab,tw. (26)

27 (agitation adj10 (bipolar or mania or manic)).ti,ab,tw. (286)

28 ("psychomotor hyperactivity" adj10 (bipolar or mania or manic)).ti,ab,tw. (1)

29 ("mani* with psychos*" or "mani* without psychos*").ti,ab,tw. (26)

30 or/13-29 (154675)

31 12 and 30 (224)

Search Name: **CENTRAL**

Date Run: 29/04/2023 14:11:10

ID Search Hits

#1 MeSH descriptor: [Clonazepam] explode all trees 198

#2 (clonazepam):ti,ab,kw (Word variations have been searched) 563

#3 (Klonopin or Rivotril or Paxam or Rivatril or Ravotril or Clonex or Clonotril or Kenoket or Kriadex or Lonazep or Antelepsin or Iktorivil or Linotril or Clonam):ti,ab,kw (Word variations have been searched) 26

#4 MeSH descriptor: [Bipolar Disorder] explode all trees 3586

#5 MeSH descriptor: [Affective Disorders, Psychotic] explode all trees 105

#6 MeSH descriptor: [Mania] explode all trees 167

#7 ("bipolar affective disorder" or mania or manic or "manic depression" or "cyclothym*" or "rapid cycling bipolar" or "manic depression" or "hypomania" or "affective psychosis" or "circular insanity" or "hyperthymia"):ti,ab,kw (Word variations have been searched) 4025

#8 ("acute mania" or "acute manic"):ti,ab,kw (Word variations have been searched) 576

#9 (chronic NEAR/5 (bipolar or mania or manic)):ti,ab,kw (Word variations have been searched) 146

#10 (relapse NEAR/5 (bipolar or mani*)):ti,ab,kw (Word variations have been searched) 542

#11 (schizo* NEAR/5 (mania or manic or mixed)):ti,ab,kw (Word variations have been searched) 253

#12 ("mixed mood episode" or "mixed affective state" or "dysphoric mania" or "mixed state psychosis"):ti,ab,kw (Word variations have been searched) 23

#13 ((puerperal or "postpartum" or "post-partum" or perinatal or "post-natal" or obstetric) NEAR/5 (mania or manic or mixed or psycho*)):ti,ab,kw (Word variations have been searched)940

#14 (Ultradian cycling):ti,ab,kw (Word variations have been searched) 28

#15 (agitation NEAR/10 (bipolar or mania or manic)):ti,ab,kw (Word variations have been searched)141

#16 ("psychomotor hyperactivity" NEAR/10 (bipolar or mania or manic)):ti,ab,kw (Word variations have been searched) 0

#17 ("mani* with psychos*" or "mani* without psychos*"):ti,ab,kw (Word variations have been searched) 0

#18 {or #1-#3} 567

#19 {or #4-#17} 7482

#20 #18 AND #19 in Trials 42

C: PRISMA checklist

Section/topic	#	Checklist item	Reported on page #
**TITLE**			
Title	1	Identify the report as a systematic review, meta-analysis, or both.	1
**ABSTRACT**			
Structured summary	2	Provide a structured summary including, as applicable: background; objectives; data sources; study eligibility criteria, participants, and interventions; study appraisal and synthesis methods; results; limitations; conclusions and implications of key findings; systematic review registration number.	1
**INTRODUCTION**			
Rationale	3	Describe the rationale for the review in the context of what is already known.	1-3
Objectives	4	Provide an explicit statement of questions being addressed with reference to participants, interventions, comparisons, outcomes, and study design (PICOS).	3-4
**METHODS**			
Protocol and registration	5	Indicate if a review protocol exists, if and where it can be accessed (e.g., Web address), and, if available, provide registration information including registration number.	3, Suppl. A
Eligibility criteria	6	Specify study characteristics (e.g., PICOS, length of follow-up) and report characteristics (e.g., years considered, language, publication status) used as criteria for eligibility, giving rationale.	3-4, Suppl. A and B
Information sources	7	Describe all information sources (e.g., databases with dates of coverage, contact with study authors to identify additional studies) in the search and date last searched.	3-4, Suppl. B and Suppl. D
Search	8	Present full electronic search strategy for at least one database, including any limits used, such that it could be repeated.	Suppl. B
Study selection	9	State the process for selecting studies (i.e., screening, eligibility, included in systematic review, and, if applicable, included in the meta-analysis).	3-4, Suppl. A
Data collection process	10	Describe method of data extraction from reports (e.g., piloted forms, independently, in duplicate) and any processes for obtaining and confirming data from investigators.	4, Suppl. A
Data items	11	List and define all variables for which data were sought (e.g., PICOS, funding sources) and any assumptions and simplifications made.	3-4, Suppl. A
Risk of bias in individual studies	12	Describe methods used for assessing risk of bias of individual studies (including specification of whether this was done at the study or outcome level), and how this information is to be used in any data synthesis.	4-5, Suppl. A
Summary measures	13	State the principal summary measures (e.g., risk ratio, difference in means).	4- 5, Suppl. A

Synthesis of results	14	Describe the methods of handling data and combining results of studies, if done, including measures of consistency (e.g., I^2) for each meta-analysis.	5, Suppl. A
Risk of bias across studies	15	Specify any assessment of risk of bias that may affect the cumulative evidence (e.g., publication bias, selective reporting within studies).	7
Additional analyses	16	Describe methods of additional analyses (e.g., sensitivity or subgroup analyses, meta-regression), if done, indicating which were pre-specified.	5, Suppl. A
**RESULTS**			
Study selection	17	Give numbers of studies screened, assessed for eligibility, and included in the review, with reasons for exclusions at each stage, ideally with a flow diagram.	5, Suppl. E and Suppl. F
Study characteristics	18	For each study, present characteristics for which data were extracted (e.g., study size, PICOS, follow-up period) and provide the citations.	5-6, Suppl. E
Risk of bias within studies	19	Present data on risk of bias of each study and, if available, any outcome level assessment (see item 12).	6, 8, 11, Suppl. G
Results of individual studies	20	For all outcomes considered (benefits or harms), present, for each study: (a) simple summary data for each intervention group (b) effect estimates and confidence intervals, ideally with a forest plot.	5-10, Suppl. H
Synthesis of results	21	Present results of each meta-analysis done, including confidence intervals and measures of consistency.	5-10, Suppl. H
Risk of bias across studies	22	Present results of any assessment of risk of bias across studies (see Item 15).	6, 8, 11, Suppl. G
Additional analysis	23	Give results of additional analyses, if done (e.g., sensitivity or subgroup analyses, meta-regression [see Item 16]).	7
**DISCUSSION**			
Summary of evidence	24	Summarize the main findings including the strength of evidence for each main outcome; consider their relevance to key groups (e.g., healthcare providers, users, and policy makers).	10-12
Limitations	25	Discuss limitations at study and outcome level (e.g., risk of bias), and at review-level (e.g., incomplete retrieval of identified research, reporting bias).	10-12
Conclusions	26	Provide a general interpretation of the results in the context of other evidence, and implications for future research.	10-12
**FUNDING**			
Funding	27	Describe sources of funding for the systematic review and other support (e.g., supply of data); role of funders for the systematic review.	13

D. Figure S1: PRISMA flow diagram

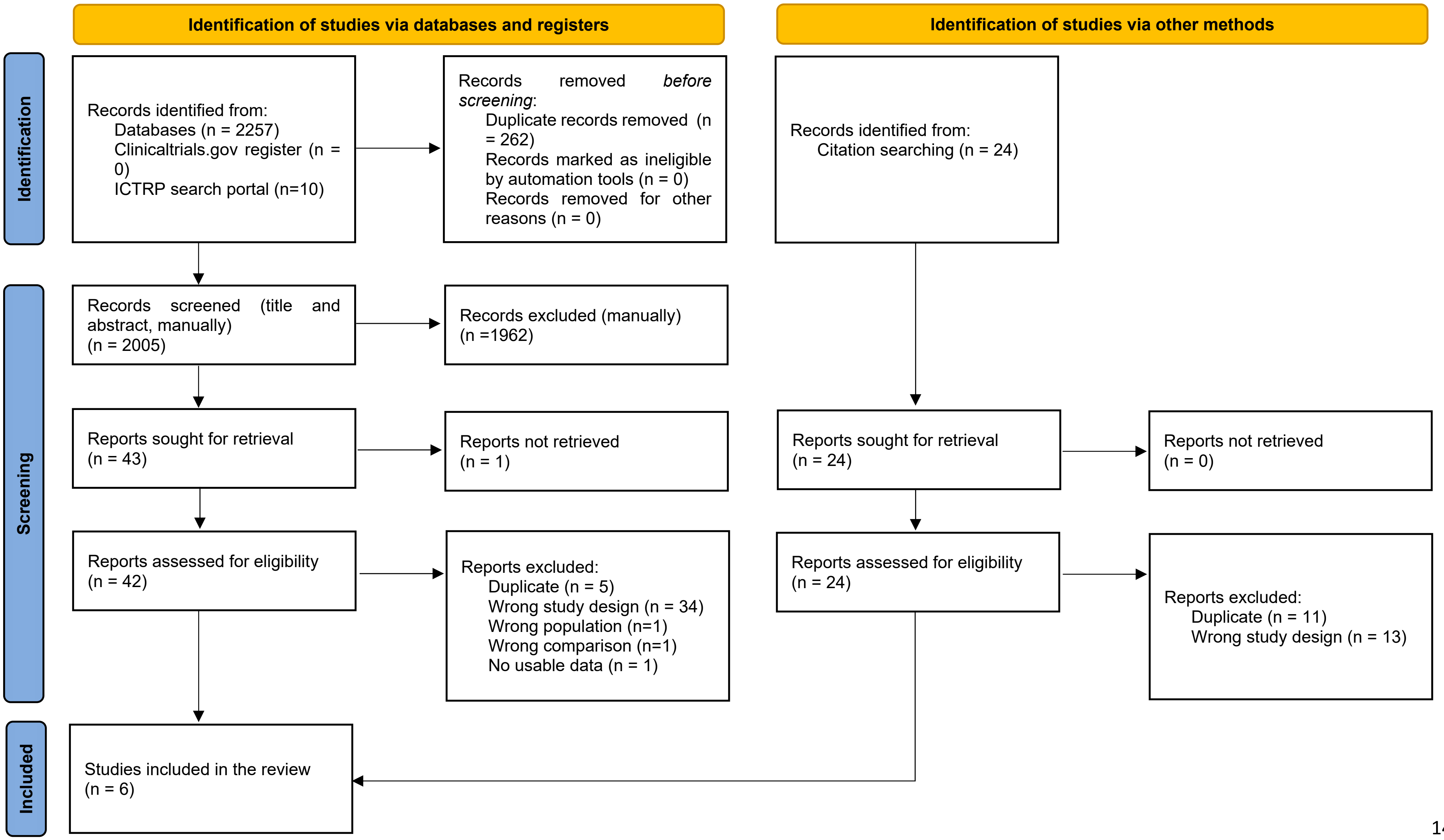

E. Table S1: List of excluded controlled studies and studies awaiting assessment

Excluded controlled studies		
**Citation**	**Reason for Exclusion**	**Comments**
Gouliaev G, Licht RW, Vestergaard P, Merinder L, Lund H, Bjerre L. Treatment of manic episodes: zuclopenthixol and clonazepam versus lithium and clonazepam. Acta Psychiatr Scand. 1996;93(2):119-24.	Wrong comparison	Comparing Zuclopenthixol vs. Lithium as add-on treatment to Clonazepam (clonazepam common in both groups)
Gouliaev G, Licht RW, Vestergaard P. Treatment of acute mania with lithium and clonazepam or zuclopenthixol and clonazepam. Clin Neuropharmacol. 1992;15(Suppl. 1):210B	Duplicate/Wrong comparison	Duplicate of the Gouliaev 1996 study above
Sachs GS, Weilburg JB, Rosenbaum JF. Clonazepam vs. neuroleptics as adjuncts to lithium maintenance. Psychopharmacol Bull. 1990;26(1):137-43.	Wrong population	Only 1 patient with current episode of mania included (relapse prevention study)
Clark H, Brook S, Alwood C. Comparative study of the efficacy of clonazepam and lithium in the treatment of acute mania. Eur Neuropsychopharmacol. 1995;0(0):378-9.	Duplicate	Duplicate of the included Clark 1997 study (preliminary results presented in conference)
Chouinard G. Antimanic effects of clonazepam. Psychosomatics. 1985;26(12 Suppl):7-12.	Duplicate	Duplicate of the included Chouinard 1983 study. Presents the same results (cross-checked for any extra data)
Benazzi F, Mazzoli M. Rapid tranquilization with intramuscular clonazepam. Can J Psychiatry. 1994;39(7):451	Duplicate	Duplicate of the included Chouinard 1993 study, no extra usable data
Dighe MS, Shrivastava AK, Trivedi JK, Desai NG. Clonazepam versus carbamazepine as add-on to lithium in acute mania. XII world congress of psychiatry, aug 24-9, 2002, yokohama, japan. 2002.	No usable data	Conference abstract with no usable data
**Studies awaiting assesment**		
de la Gándara Martín JJ, de Dios Francos A, López Gómez I, Hernández Herrero H, Redondo Martínez AL. Tratamiento de trastornos afectivos con clonazepan [Treatment of affective disorders with clonazepam]. Actas Luso Esp Neurol Psiquiatr Cienc Afines. 1991;19(2):88-96.	Unable to locate full-text	Sources searched: web, National Library of Medicine, British Library, authors contacted. Information in the abstract indicates that this is probably an uncontrolled study.

F: Forest Plots for all outcomes

Study or Subgroup	Clonazepam Mean	Clonazepam SD	Clonazepam Total	Placebo Mean	Placebo SD	Placebo Total	Weight	Std. Mean Difference IV, Random, 95% CI
Edwards 1991	-3.45	1.258	20	-1.925	1.178	20	100.0%	-1.23 [-1.91, -0.55]
**Total (95% CI)**			**20**			**20**	**100.0%**	**-1.23 [-1.91, -0.55]**

Heterogeneity: Not applicable
Test for overall effect: Z = 3.53 (P = 0.0004)

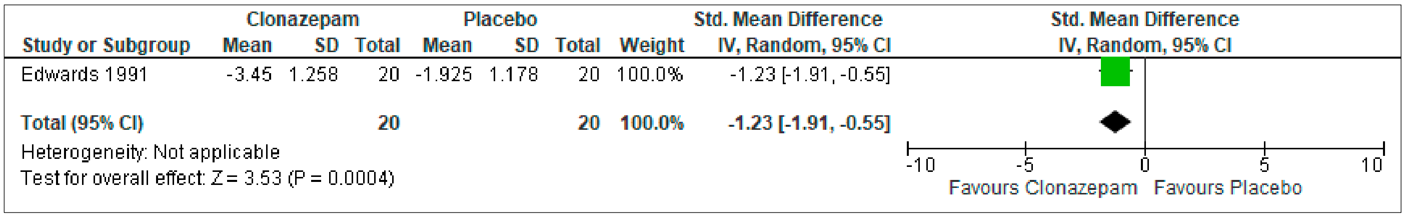

Figure S2: Clonazepam vs. Placebo. Outcome: efficacy, response to treatment (continuous)

Study or Subgroup	Experimental Mean	Experimental SD	Experimental Total	Control Mean	Control SD	Control Total	Weight	Std. Mean Difference IV, Random, 95% CI
**2.1.1 Response - acute**								
Bradwejn 1990a	4.8	1.757	11	3.3	1.757	13	100.0%	0.82 [-0.02, 1.67]
**Subtotal (95% CI)**			**11**			**13**	**100.0%**	**0.82 [-0.02, 1.67]**
Heterogeneity: Not applicable								
Test for overall effect: Z = 1.92 (P = 0.06)								
**Total (95% CI)**			**11**			**13**	**100.0%**	**0.82 [-0.02, 1.67]**

Heterogeneity: Not applicable
Test for overall effect: Z = 1.92 (P = 0.06)
Test for subgroup differences: Not applicable

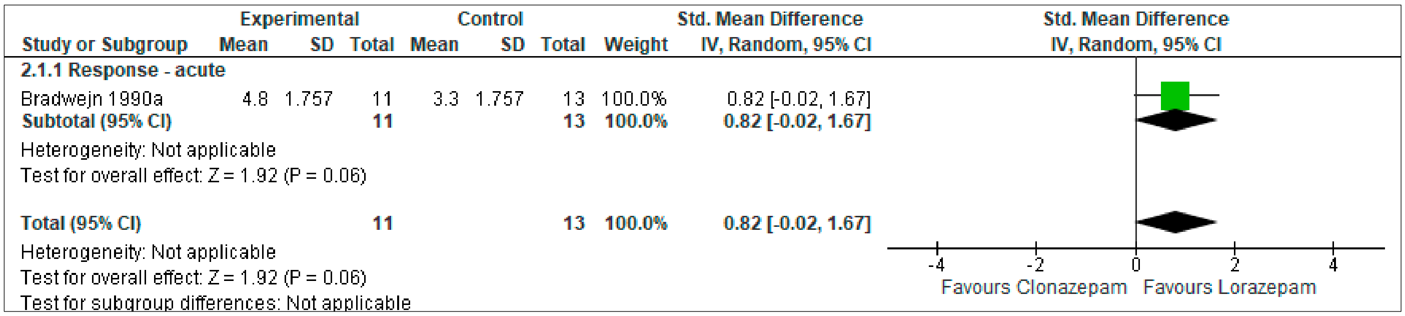

Study or Subgroup	Experimental Mean	Experimental SD	Experimental Total	Control Mean	Control SD	Control Total	Weight	Std. Mean Difference IV, Random, 95% CI
**2.1.2 Response - long-term**								
Bradwejn 1990b	3.33	1.359	10	2.17	1.359	9	100.0%	0.82 [-0.13, 1.76]
**Subtotal (95% CI)**			**10**			**9**	**100.0%**	**0.82 [-0.13, 1.76]**
Heterogeneity: Not applicable								
Test for overall effect: Z = 1.69 (P = 0.09)								
**Total (95% CI)**			**10**			**9**	**100.0%**	**0.82 [-0.13, 1.76]**

Heterogeneity: Not applicable
Test for overall effect: Z = 1.69 (P = 0.09)
Test for subgroup differences: Not applicable

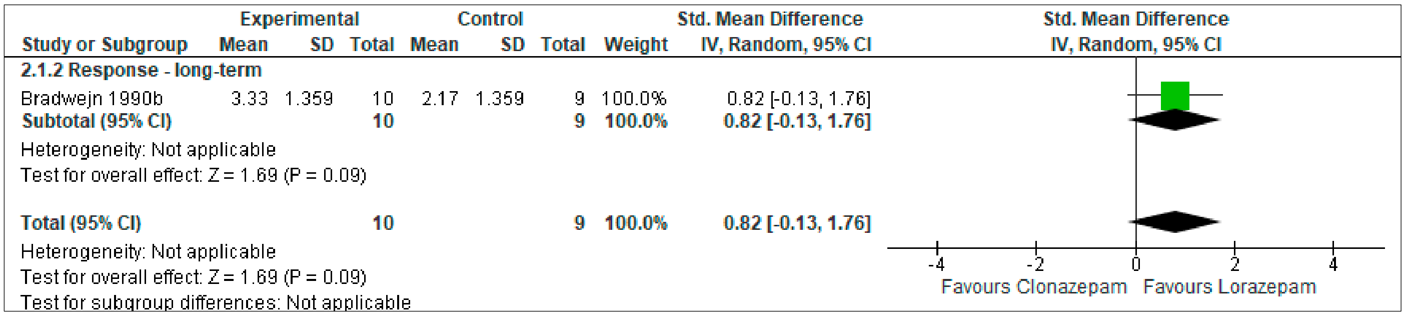

Figure S3: Clonazepam vs. Lorazepam. Outcome: efficacy, response to treatment (continuous). Bradwejn 1990a: presents the acute effects (up to 3 weeks). Bradwejn 1990b: presents the long-term effects (more than 3 weeks of continuous treatment).

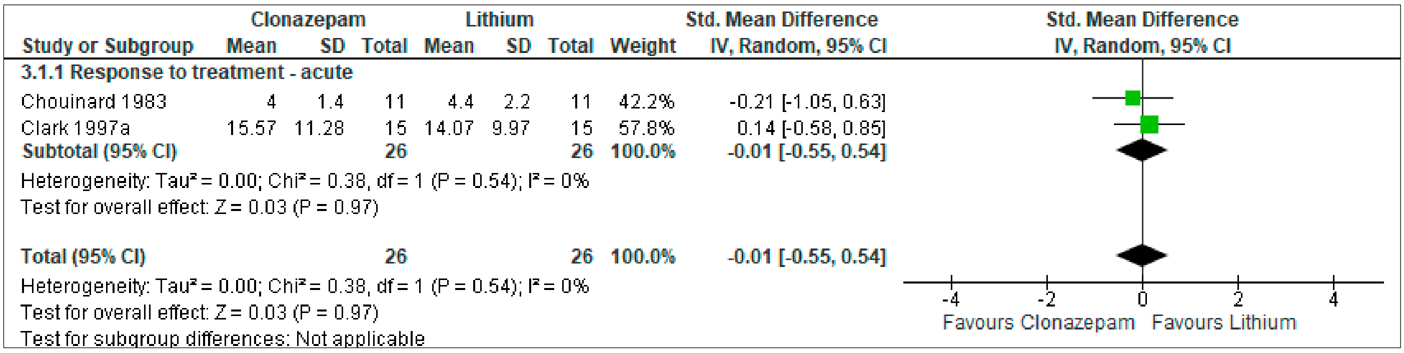

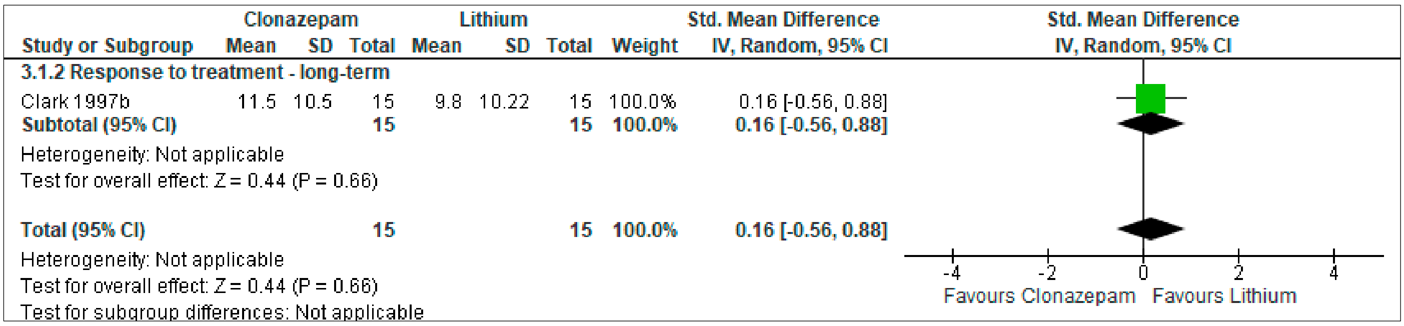

Figure S4. Clonazepam vs. Lithium. Outcome: efficacy, response to treatment (continuous). Clark 1997a: presents the acute effects (up to 3 weeks). Clark 1997b: presents the long-term effects (more than 3 weeks of continuous treatment).

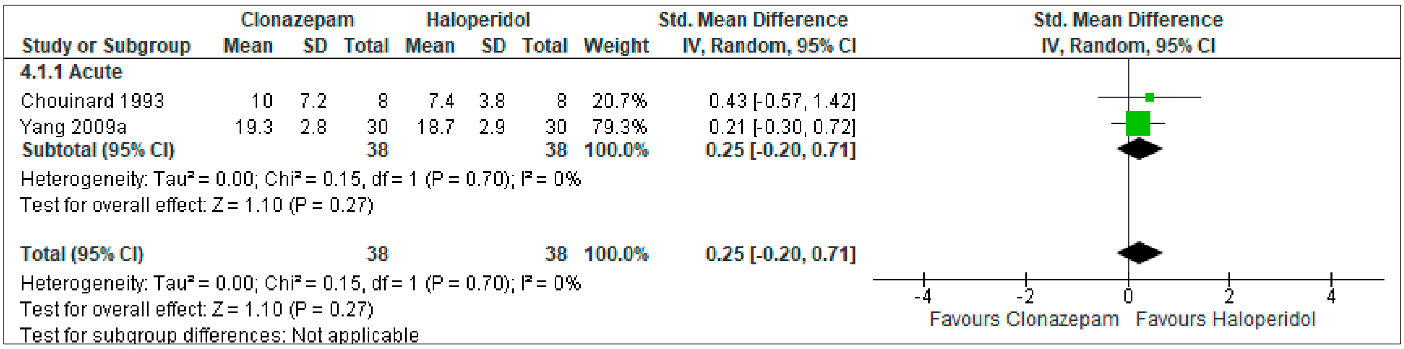

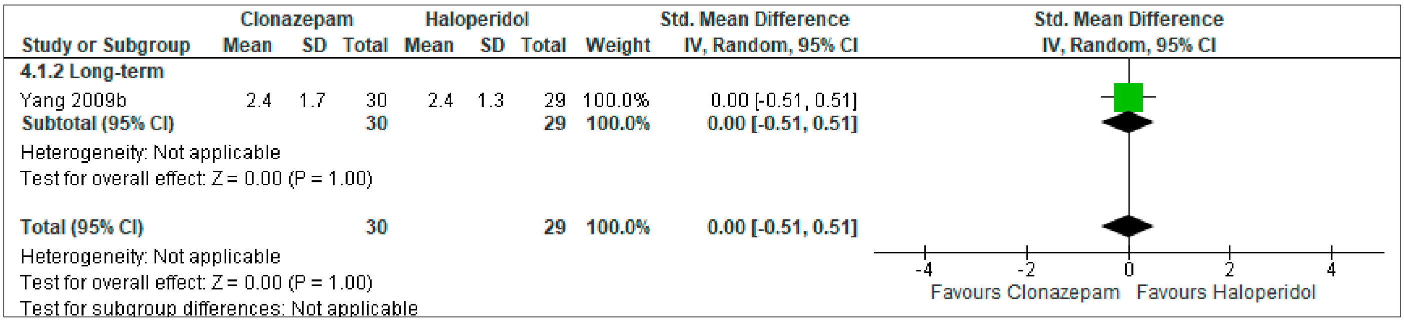

Figure S5. Clonazepam vs. Haloperidol. Outcome: efficacy, response to treatment (continuous).Yang 2009a: presents the acute effects (up to 3 weeks). Yang 2009b: presents the long-term effects (more than 3 weeks of continuous treatment).

Study or Subgroup	Clonazepam Events	Clonazepam Total	Control Events	Control Total	Weight	Odds Ratio IV, Random, 95% CI
**5.1.1 Clonazepam vs. placebo**						
Edwards 1991	0	20	0	20		Not estimable
**Subtotal (95% CI)**		**20**		**20**		**Not estimable**
Total events	0		0			
Heterogeneity: Not applicable						
Test for overall effect: Not applicable						
**5.1.2 Clonazepam vs. Lorazepam**						
Bradwejn 1990a	0	11	0	13		Not estimable
**Subtotal (95% CI)**		**11**		**13**		**Not estimable**
Total events	0		0			
Heterogeneity: Not applicable						
Test for overall effect: Not applicable						
**5.1.3 Clonazepam vs. Lithium**						
Chouinard 1983	0	11	0	11		Not estimable
Clark 1997a	0	15	0	15		Not estimable
**Subtotal (95% CI)**		**26**		**26**		**Not estimable**
Total events	0		0			
Heterogeneity: Not applicable						
Test for overall effect: Not applicable						
**5.1.4 Clonazepam vs. Haloperidol**						
Chouinard 1993	0	8	2	8	50.6%	0.15 [0.01, 3.77]
Yang 2009a	0	30	1	30	49.4%	0.32 [0.01, 8.24]
**Subtotal (95% CI)**		**38**		**38**	**100.0%**	**0.22 [0.02, 2.16]**
Total events	0		3			
Heterogeneity: Tau² = 0.00; Chi² = 0.10, df = 1 (P = 0.75); I² = 0%						
Test for overall effect: Z = 1.30 (P = 0.19)						
**Total (95% CI)**		**95**		**97**	**100.0%**	**0.22 [0.02, 2.16]**
Total events	0		3			
Heterogeneity: Tau² = 0.00; Chi² = 0.10, df = 1 (P = 0.75); I² = 0%						
Test for overall effect: Z = 1.30 (P = 0.19)						
Test for subgroup differences: Not applicable						

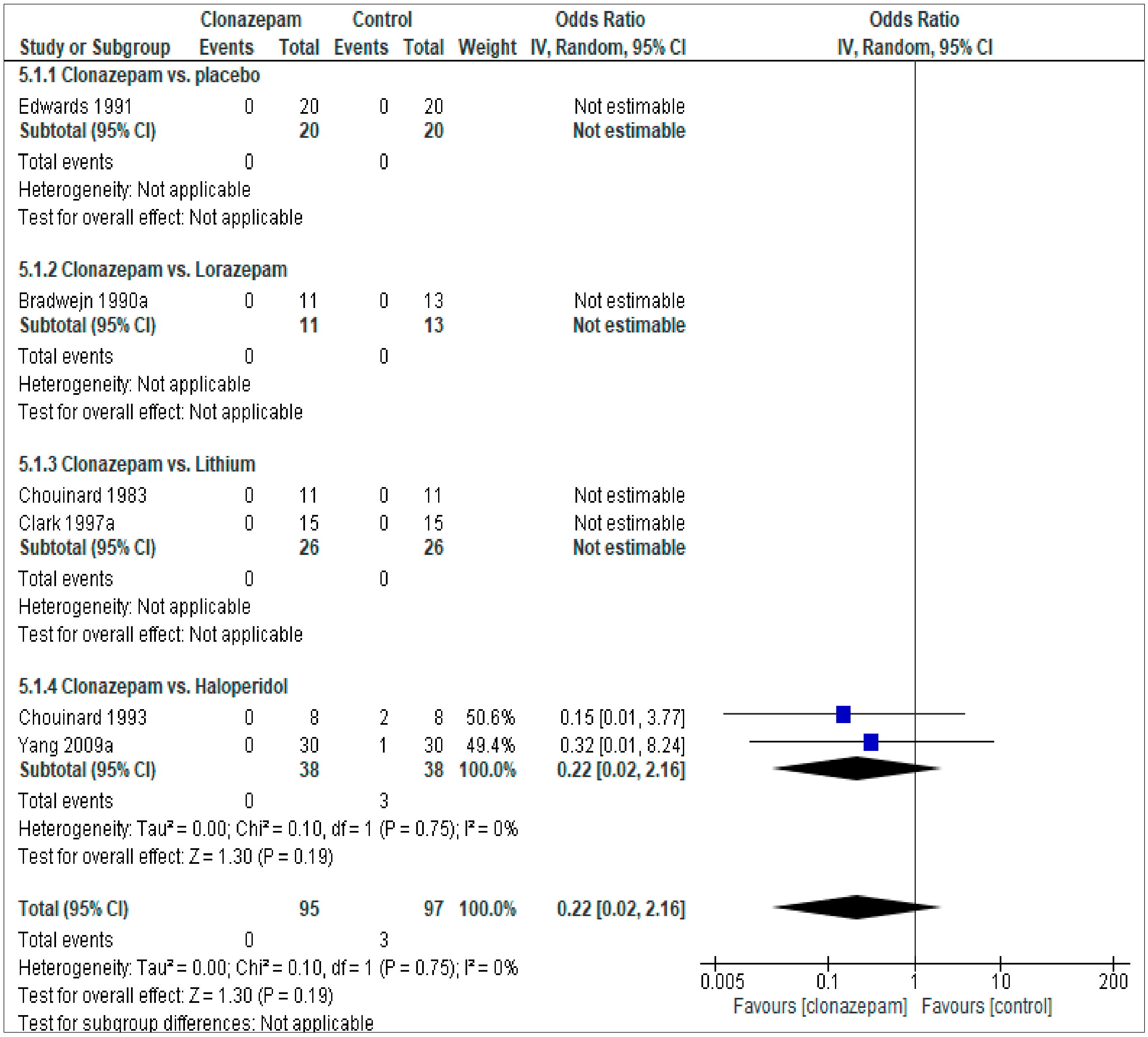

Study or Subgroup	Clonazepam Events	Clonazepam Total	Control Events	Control Total	Weight	Odds Ratio M-H, Random, 95% CI
Bradwejn 1990b	0	10	0	9		Not estimable
Clark 1997b	0	15	0	15		Not estimable
Yang 2009b	0	30	1	30	100.0%	0.32 [0.01, 8.24]
**Total (95% CI)**		**55**		**54**	**100.0%**	**0.32 [0.01, 8.24]**
Total events	0		1			
Heterogeneity: Not applicable						
Test for overall effect: Z = 0.68 (P = 0.49)						

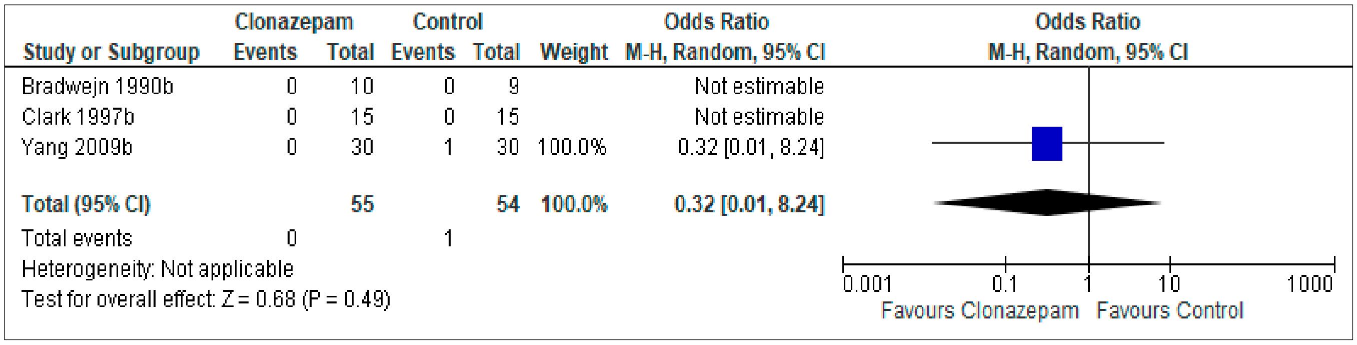

Figure S6. Tolerability (discontinuation due to adverse effects, measured as the proportion of patients who dropped out due to adverse effects): Clonazepam vs. any other pharmacotherapy, including placebo. Bradwejn 1990a, Clark 1997a and Yang 2009a: present the acute effects (up to 3 weeks). Bradwejn 1990b, Clark 1997b and Yang 2009b: present the long-term effects (more than 3 weeks of continuous treatment).

Study or Subgroup	Clonazepam Events	Clonazepam Total	Control Events	Control Total	Weight	Odds Ratio IV, Random, 95% CI
**6.1.1 Clonazepam vs. placebo**						
Edwards 1991	0	20	0	20		Not estimable
**Subtotal (95% CI)**		**20**		**20**		**Not estimable**
Total events	0		0			
Heterogeneity: Not applicable						
Test for overall effect: Not applicable						
**6.1.2 Clonazepam vs. Lorazepam**						
Bradwejn 1990a	1	11	2	13	35.3%	0.55 [0.04, 7.03]
**Subtotal (95% CI)**		**11**		**13**	**35.3%**	**0.55 [0.04, 7.03]**
Total events	1		2			
Heterogeneity: Not applicable						
Test for overall effect: Z = 0.46 (P = 0.65)						
**6.1.3 Clonazepam vs. Lithium**						
Chouinard 1983	0	11	1	11	21.0%	0.30 [0.01, 8.32]
Clark 1997a	1	15	0	15	21.3%	3.21 [0.12, 85.20]
**Subtotal (95% CI)**		**26**		**26**	**42.3%**	**1.00 [0.10, 10.25]**
Total events	1		1			
Heterogeneity: Tau² = 0.00; Chi² = 0.98, df = 1 (P = 0.32); I² = 0%						
Test for overall effect: Z = 0.00 (P = 1.00)						
**6.1.4 Clonazepam vs. Haloperidol**						
Chouinard 1993	0	8	2	8	22.4%	0.15 [0.01, 3.77]
Yang 2009a	0	30	0	30		Not estimable
**Subtotal (95% CI)**		**38**		**38**	**22.4%**	**0.15 [0.01, 3.77]**
Total events	0		2			
Heterogeneity: Not applicable						
Test for overall effect: Z = 1.15 (P = 0.25)						
**Total (95% CI)**		**95**		**97**	**100.0%**	**0.53 [0.12, 2.42]**
Total events	2		5			
Heterogeneity: Tau² = 0.00; Chi² = 1.84, df = 3 (P = 0.61); I² = 0%						
Test for overall effect: Z = 0.82 (P = 0.41)						
Test for subgroup differences: Chi² = 0.86, df = 2 (P = 0.65), I² = 0%						

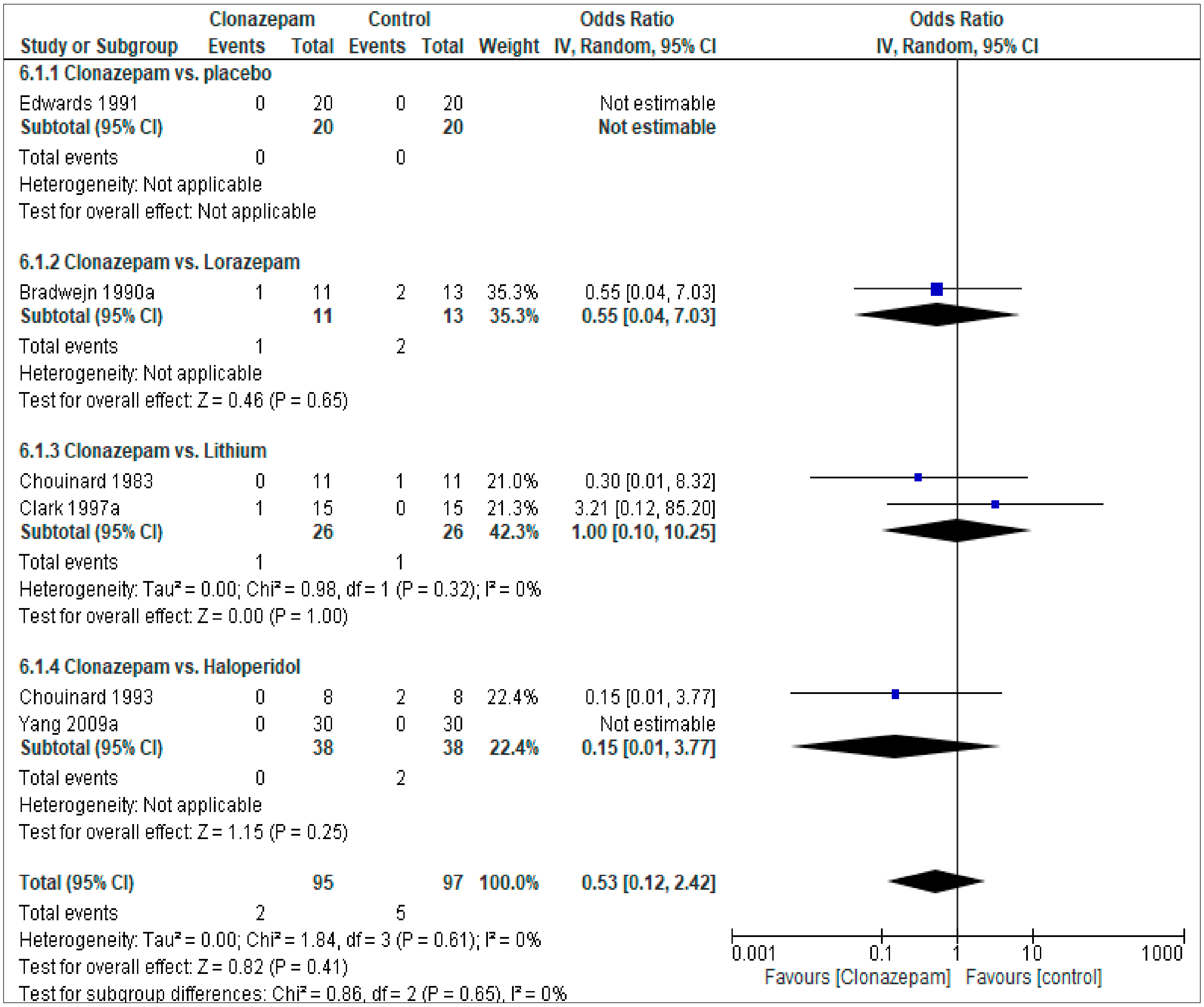

Study or Subgroup	Clonazepam Events	Clonazepam Total	Control Events	Control Total	Weight	Odds Ratio IV, Random, 95% CI
Bradwejn 1990b	1	10	4	9	45.1%	0.14 [0.01, 1.61]
Clark 1997b	1	15	0	15	27.2%	3.21 [0.12, 85.20]
Yang 2009b	0	30	1	30	27.8%	0.32 [0.01, 8.24]
**Total (95% CI)**		**55**		**54**	**100.0%**	**0.41 [0.07, 2.51]**
Total events	2		5			
Heterogeneity: Tau² = 0.32; Chi² = 2.28, df = 2 (P = 0.32); I² = 12%						
Test for overall effect: Z = 0.96 (P = 0.34)						

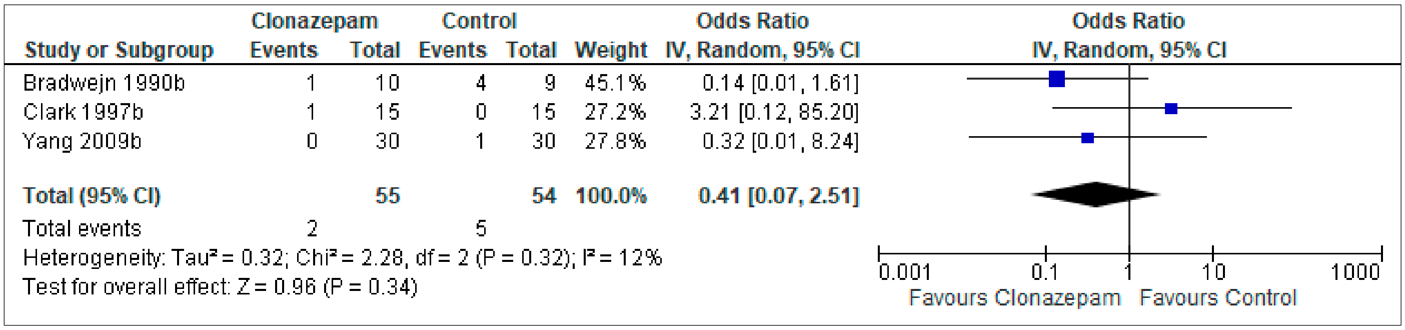

Figure S7. Acceptability (all cause discontinuation, measured as the proportion of patients who dropped out due to any reason): Clonazepam vs. any other pharmacotherapy, including placebo. Bradwejn 1990a, Clark 1997a and Yang 2009a: present the acute effects (up to 3 weeks). Bradwejn 1990b, Clark 1997b and Yang 2009b: present the medium and long-term effects (more than 3 weeks of continuous treatment).

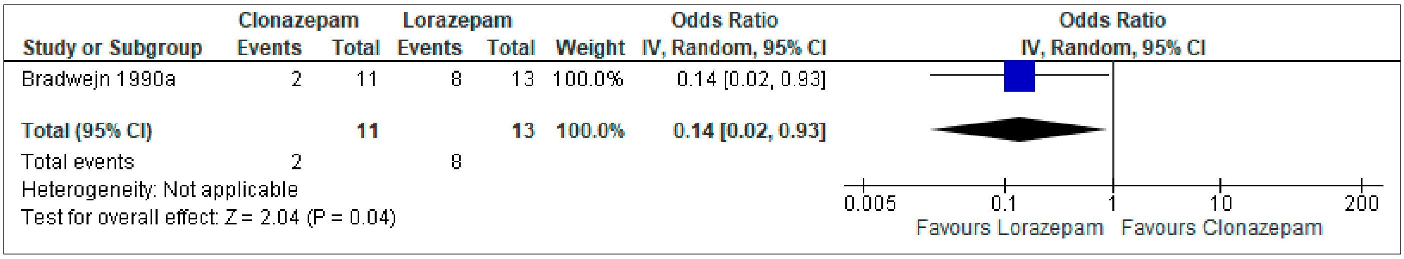

Figure S8: Clonazepam vs. Lorazepam. Response to treatment (dichotomous), in the acute phase (up to 3 weeks of treatment).

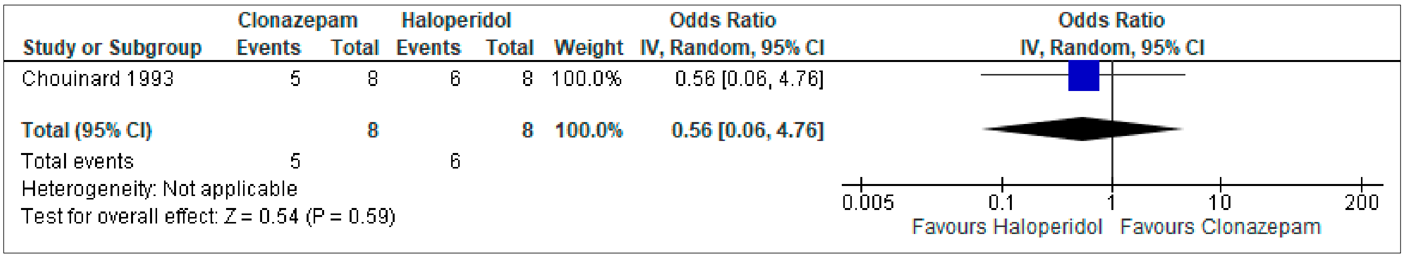

Figure S9: Clonazepam vs. Haloperidol. Response to treatment (dichotomous), in the acute phase (rapid tranquilization in this particular study).

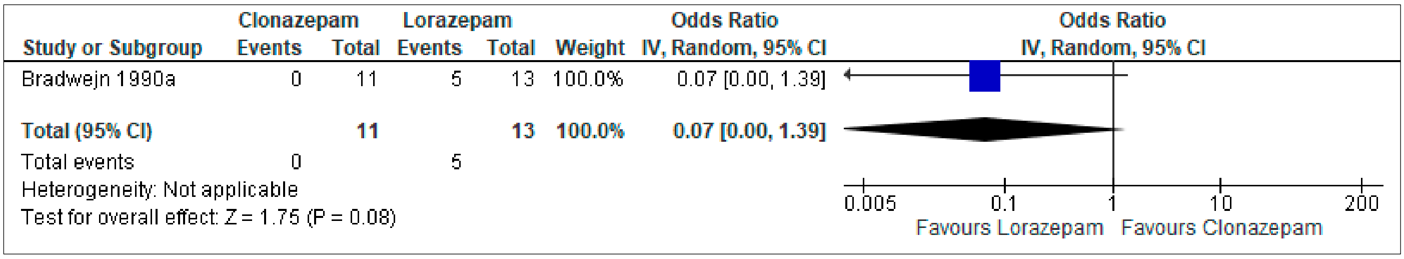

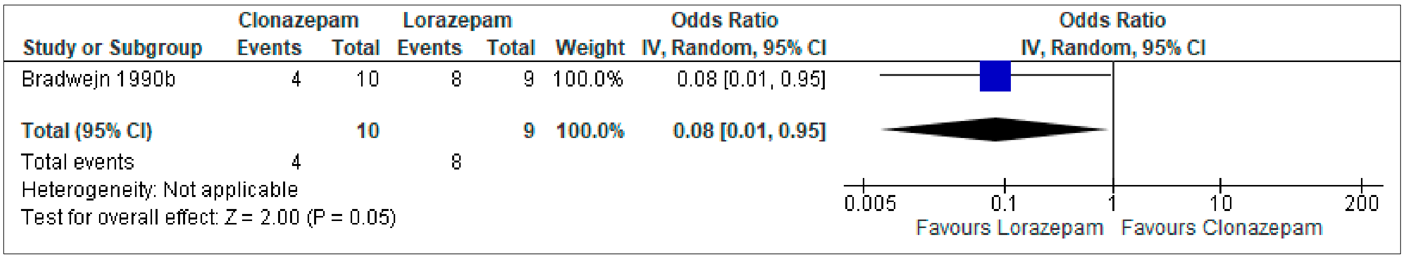

Figure S10: Clonazepam vs. Lorazepam. Outcome: remission (dichotomous). Bradwejn 1990a: presents the acute effects (up to 3 weeks). Bradwejn 1990b: presents the long-term effects (more than 3 weeks of continuous treatment).

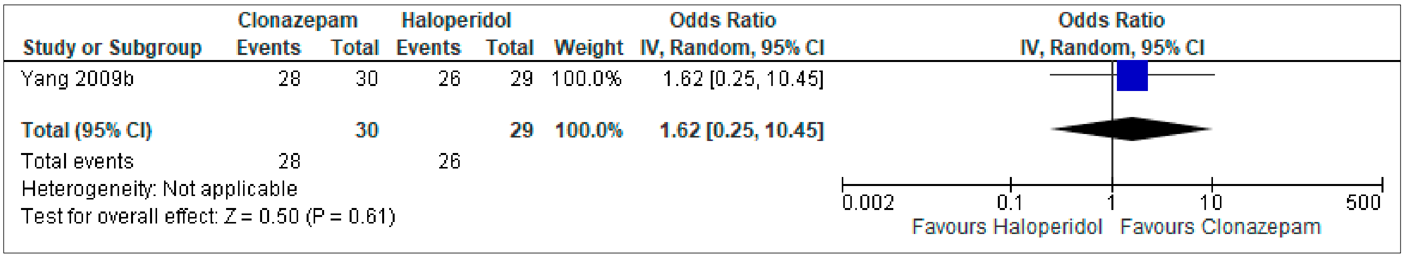

Figure S11: Clonazepam vs. Haloperidol. Outcome: remission (dichotomous). Presents the long-term effects (16 weeks).

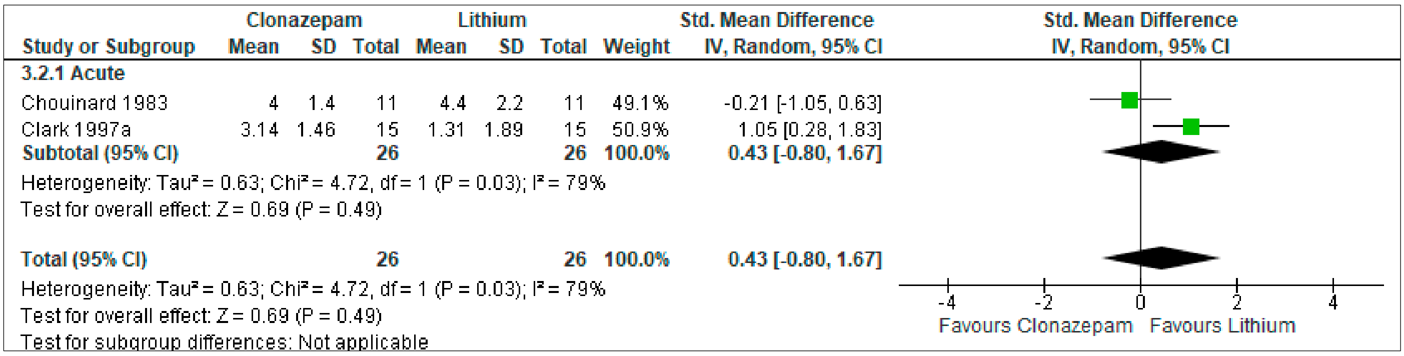

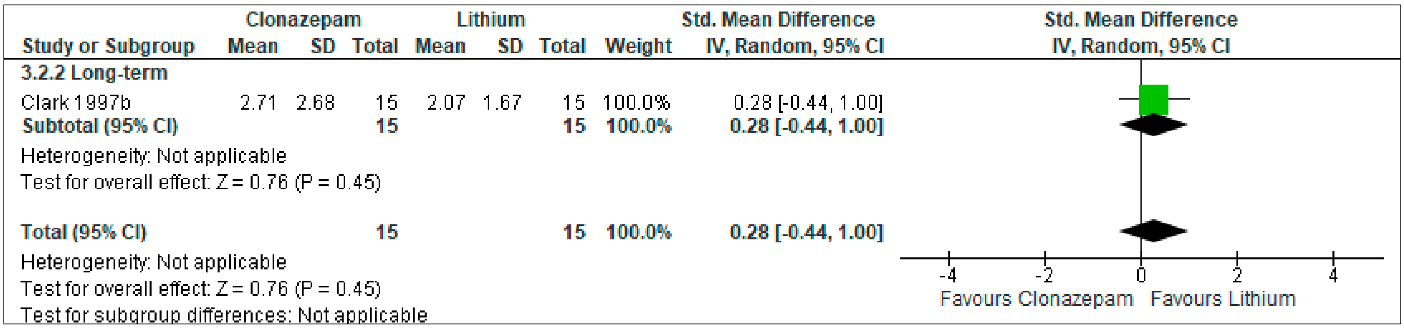

Figure S12: Clonazepam vs. Lithium. Outcome: efficacy, global state (continuous, using the Clinical Global Impression Scale). Clark 1997a: presents the acute effects (up to 3 weeks). Clark 1997b: presents the long-term effects (more than 3 weeks of continuous treatment).

Study or Subgroup	Clonazepam Mean	SD	Total	Haloperidol Mean	SD	Total	Weight	Std. Mean Difference IV, Random, 95% CI
**4.4.1 Acute**								
Chouinard 1993	2.2	1.3	8	2.6	1.3	8	34.4%	-0.29 [-1.28, 0.70]
Yang 2009a	4.3	0.5	30	4.1	0.3	30	65.6%	0.48 [-0.03, 0.99]
**Subtotal (95% CI)**			**38**			**38**	**100.0%**	**0.21 [-0.50, 0.93]**
Heterogeneity: Tau² = 0.14; Chi² = 1.84, df = 1 (P = 0.18); I² = 46%								
Test for overall effect: Z = 0.59 (P = 0.56)								
**Total (95% CI)**			**38**			**38**	**100.0%**	**0.21 [-0.50, 0.93]**
Heterogeneity: Tau² = 0.14; Chi² = 1.84, df = 1 (P = 0.18); I² = 46%								
Test for overall effect: Z = 0.59 (P = 0.56)								
Test for subgroup differences: Not applicable								

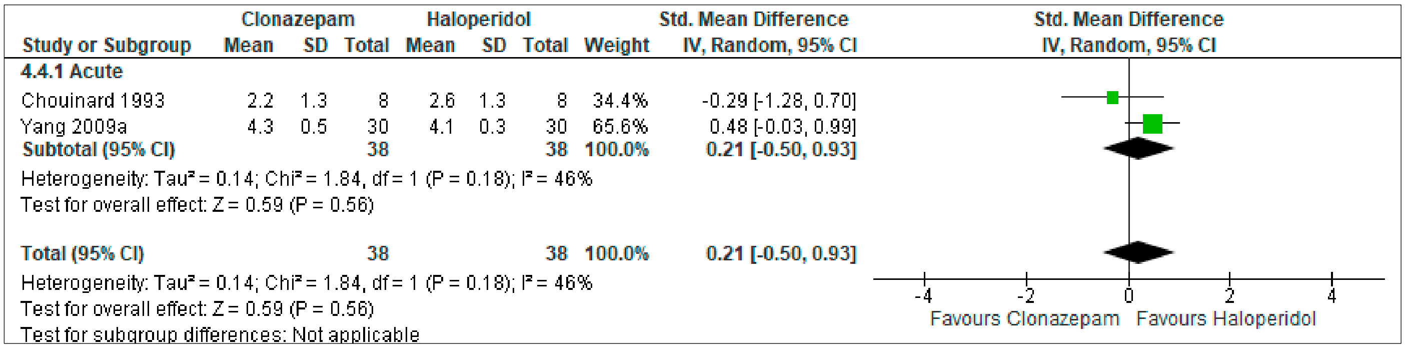

Study or Subgroup	Clonazepam Mean	SD	Total	Haloperidol Mean	SD	Total	Weight	Std. Mean Difference IV, Random, 95% CI
**4.4.2 Long-term**								
Yang 2009b	1.7	0.4	30	1.5	0.5	29	100.0%	0.44 [-0.08, 0.95]
**Subtotal (95% CI)**			**30**			**29**	**100.0%**	**0.44 [-0.08, 0.95]**
Heterogeneity: Not applicable								
Test for overall effect: Z = 1.66 (P = 0.10)								
**Total (95% CI)**			**30**			**29**	**100.0%**	**0.44 [-0.08, 0.95]**
Heterogeneity: Not applicable								
Test for overall effect: Z = 1.66 (P = 0.10)								
Test for subgroup differences: Not applicable								

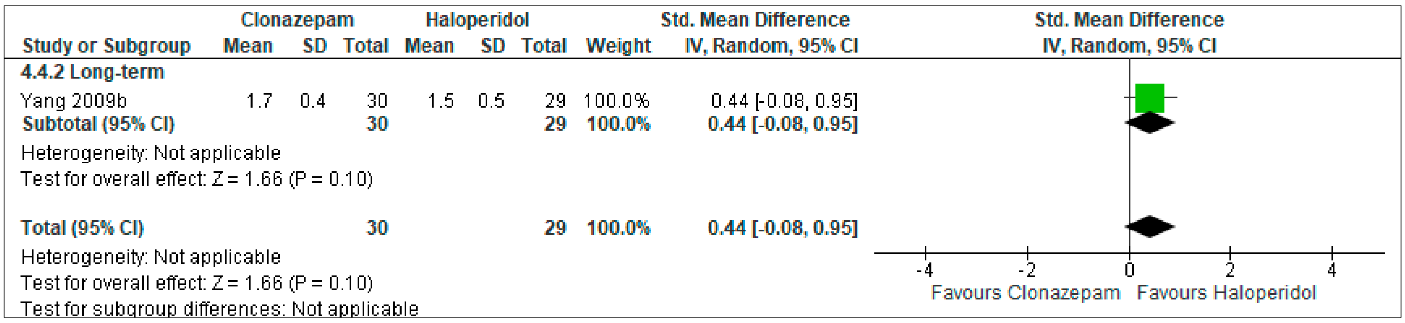

Figure S13: Clonazepam vs. Haloperidol. Outcome: efficacy, global state (continuous, using the Clinical Global Impression Scale). Yang 2009a: presents the acute effects (up to 3 weeks). Yang 2009b: presents the long-term effects (more than 3 weeks of continuous treatment).

Study or Subgroup	Clonazepam Mean	SD	Total	Lithium Mean	SD	Total	Weight	Std. Mean Difference IV, Random, 95% CI
**3.3.1 Acute**								
Clark 1997a	-45	21.84	15	-46.33	15.98	15	100.0%	0.07 [-0.65, 0.78]
**Subtotal (95% CI)**			**15**			**15**	**100.0%**	**0.07 [-0.65, 0.78]**
Heterogeneity: Not applicable								
Test for overall effect: Z = 0.19 (P = 0.85)								
**Total (95% CI)**			**15**			**15**	**100.0%**	**0.07 [-0.65, 0.78]**
Heterogeneity: Not applicable								
Test for overall effect: Z = 0.19 (P = 0.85)								
Test for subgroup differences: Not applicable								

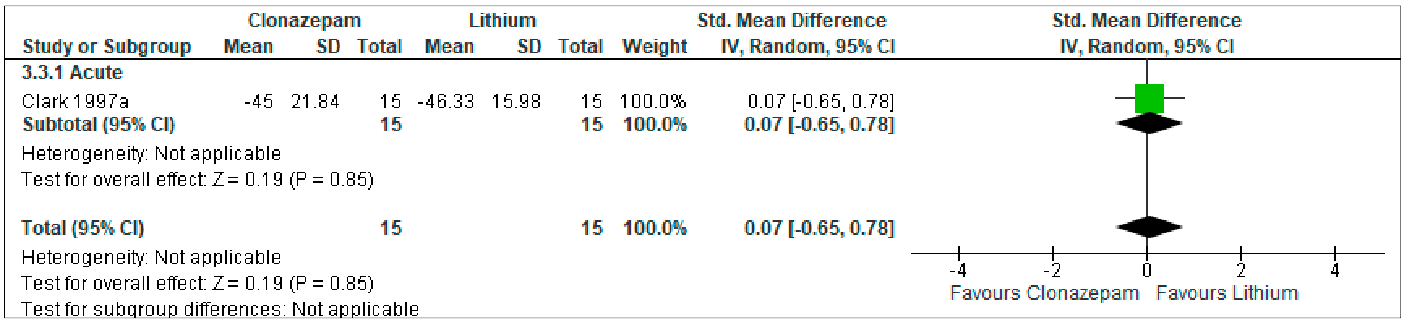

Study or Subgroup	Clonazepam Mean	SD	Total	Lithium Mean	SD	Total	Weight	Std. Mean Difference IV, Random, 95% CI
**3.3.2 Long-term**								
Clark 1997b	-54.29	20.56	15	-44	24	15	100.0%	-0.45 [-1.17, 0.28]
**Subtotal (95% CI)**			**15**			**15**	**100.0%**	**-0.45 [-1.17, 0.28]**
Heterogeneity: Not applicable								
Test for overall effect: Z = 1.21 (P = 0.23)								
**Total (95% CI)**			**15**			**15**	**100.0%**	**-0.45 [-1.17, 0.28]**
Heterogeneity: Not applicable								
Test for overall effect: Z = 1.21 (P = 0.23)								
Test for subgroup differences: Not applicable								

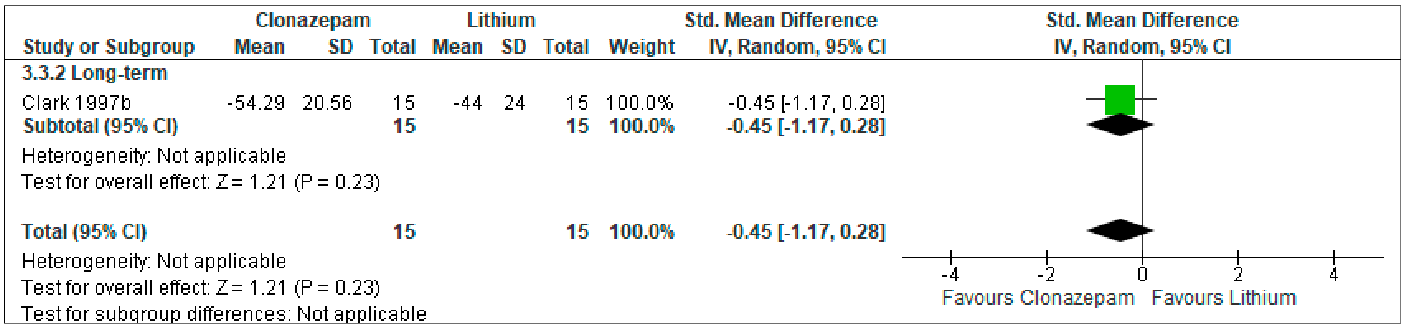

Figure S14: Clonazepam vs. Lithium. Outcome: functioning (continuous). Clark 1997a: presents the acute effects (up to 3 weeks). Clark 1997b: presents the long-term effects (more than 3 weeks of continuous treatment).

Study or Subgroup	Clonazepam Mean	Clonazepam SD	Clonazepam Total	Haloperidol Mean	Haloperidol SD	Haloperidol Total	Weight	Std. Mean Difference IV, Random, 95% CI
Yang 2009b	-77.1	3	30	-77.3	4.7	29	100.0%	0.05 [-0.46, 0.56]
**Total (95% CI)**			**30**			**29**	**100.0%**	**0.05 [-0.46, 0.56]**
Heterogeneity: Not applicable								
Test for overall effect: Z = 0.19 (P = 0.85)								

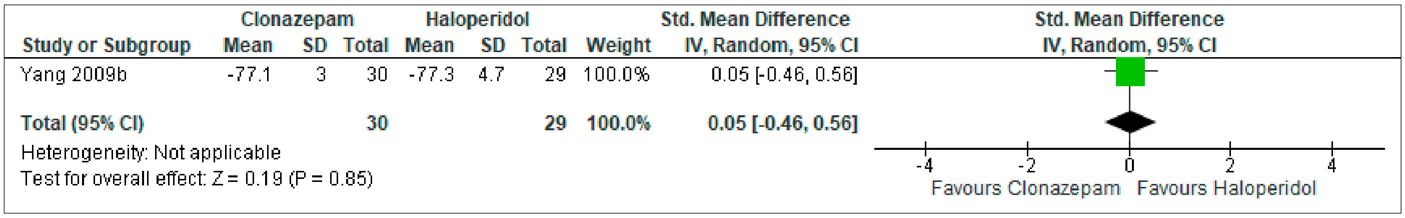

Figure S15: Clonazepam vs. Haloperidol. Outcome: functioning (continuous). Yang 2009b: presents the long-term effects (more than 3 weeks of continuous treatment).

Study or Subgroup	Clonazepam Mean	Clonazepam SD	Clonazepam Total	Placebo Mean	Placebo SD	Placebo Total	Weight	Std. Mean Difference IV, Random, 95% CI
Edwards 1991	-1.225	1.686	20	-0.918	1.472	20	100.0%	-0.19 [-0.81, 0.43]
**Total (95% CI)**			**20**			**20**	**100.0%**	**-0.19 [-0.81, 0.43]**
Heterogeneity: Not applicable								
Test for overall effect: Z = 0.60 (P = 0.55)								

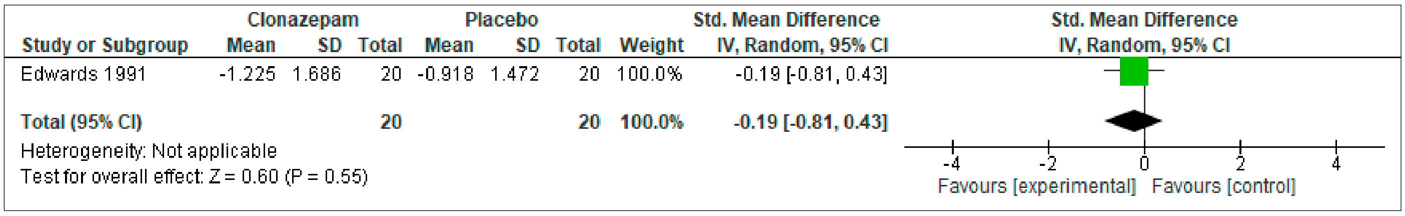

Figure S16: Clonazepam vs. Placebo. Outcome: efficacy, psychotic symptoms (continuous)

Study or Subgroup	Clonazepam Mean	Clonazepam SD	Clonazepam Total	Lithium Mean	Lithium SD	Lithium Total	Weight	Std. Mean Difference IV, Random, 95% CI
**3.4.1 Acute**								
Clark 1997a	11.64	10.4	15	7.07	6.35	15	100.0%	0.52 [-0.21, 1.25]
**Subtotal (95% CI)**			**15**			**15**	**100.0%**	**0.52 [-0.21, 1.25]**
Heterogeneity: Not applicable								
Test for overall effect: Z = 1.39 (P = 0.17)								
**Total (95% CI)**			**15**			**15**	**100.0%**	**0.52 [-0.21, 1.25]**
Heterogeneity: Not applicable								
Test for overall effect: Z = 1.39 (P = 0.17)								
Test for subgroup differences: Not applicable								

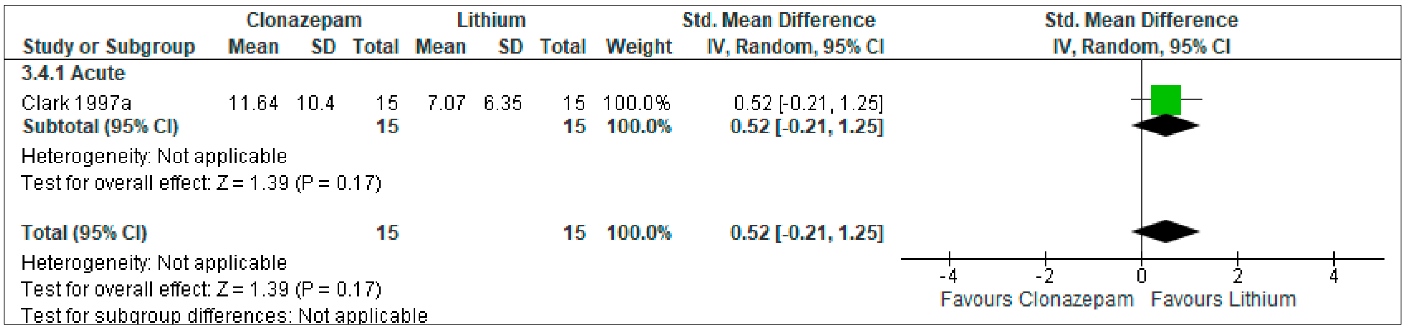

Study or Subgroup	Clonazepam Mean	Clonazepam SD	Clonazepam Total	Lithium Mean	Lithium SD	Lithium Total	Weight	Std. Mean Difference IV, Random, 95% CI
**3.4.2 Long-term**								
Clark 1997b	7.79	6.89	15	6.27	7.52	15	100.0%	0.21 [-0.51, 0.92]
**Subtotal (95% CI)**			**15**			**15**	**100.0%**	**0.21 [-0.51, 0.92]**
Heterogeneity: Not applicable								
Test for overall effect: Z = 0.56 (P = 0.58)								
**Total (95% CI)**			**15**			**15**	**100.0%**	**0.21 [-0.51, 0.92]**
Heterogeneity: Not applicable								
Test for overall effect: Z = 0.56 (P = 0.58)								
Test for subgroup differences: Not applicable								

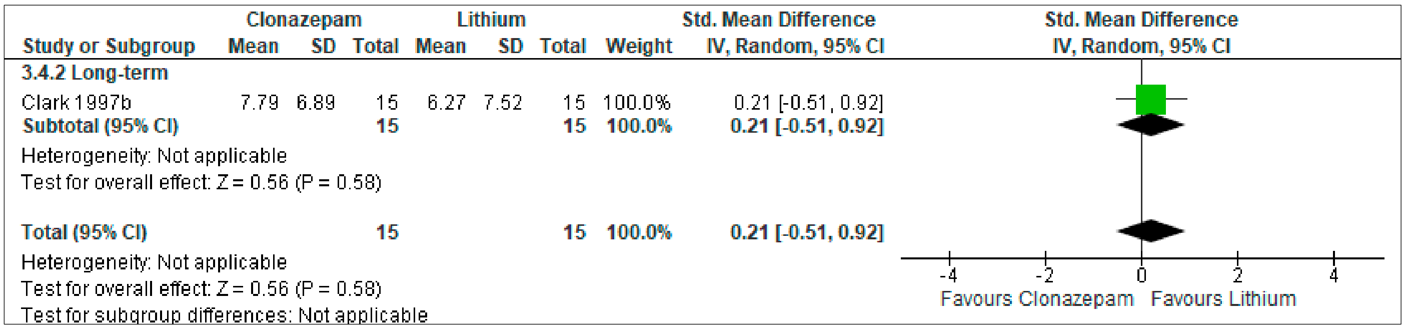

Figure S17: Clonazepam vs. Lithium. Outcome: efficacy, psychotic symptoms (continuous). Clark 1997a: presents the acute effects (up to 3 weeks). Clark 1997b: presents the long-term effects (more than 3 weeks of continuous treatment).

Study or Subgroup	Clonazepam Mean	Clonazepam SD	Clonazepam Total	Haloperidol Mean	Haloperidol SD	Haloperidol Total	Weight	Std. Mean Difference IV, Random, 95% CI
Chouinard 1993	1.3	1.6	8	1.7	1.6	8	100.0%	-0.24 [-1.22, 0.75]
**Total (95% CI)**			**8**			**8**	**100.0%**	**-0.24 [-1.22, 0.75]**
Heterogeneity: Not applicable								
Test for overall effect: Z = 0.47 (P = 0.64)								

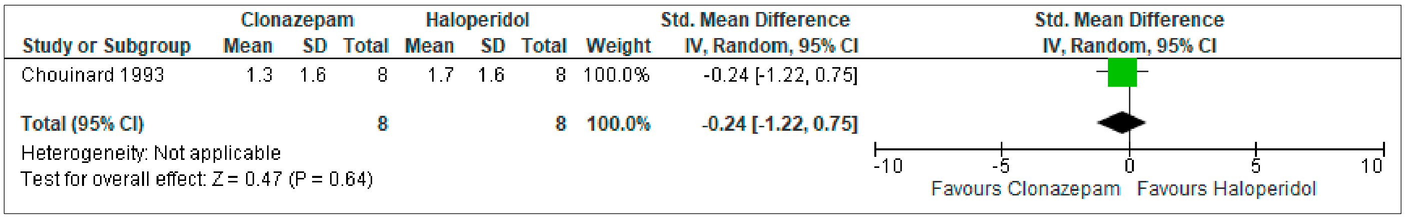

Figure S18: Clonazepam vs. Haloperidol. Outcome: efficacy, psychotic symptoms (continuous).

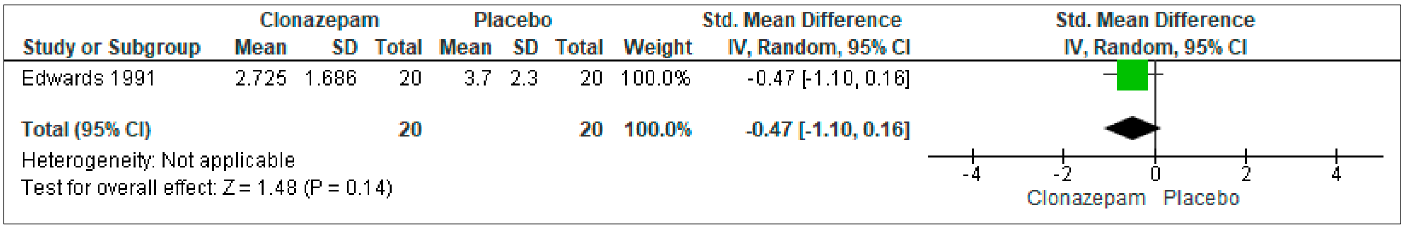

Figure S19: Clonazepam vs. Placebo. Outcome: total antipsychotic dose over the course of treatment (continuous, chlorpromazine, measured in grams).

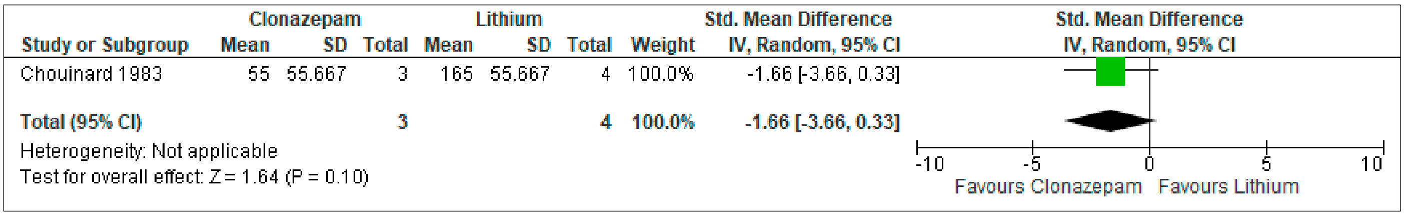

Figure S20: Clonazepam vs. Lithium. Outcome: total antipsychotic dose over the course of treatment (continuous, Haloperidol, measured in milligrams).

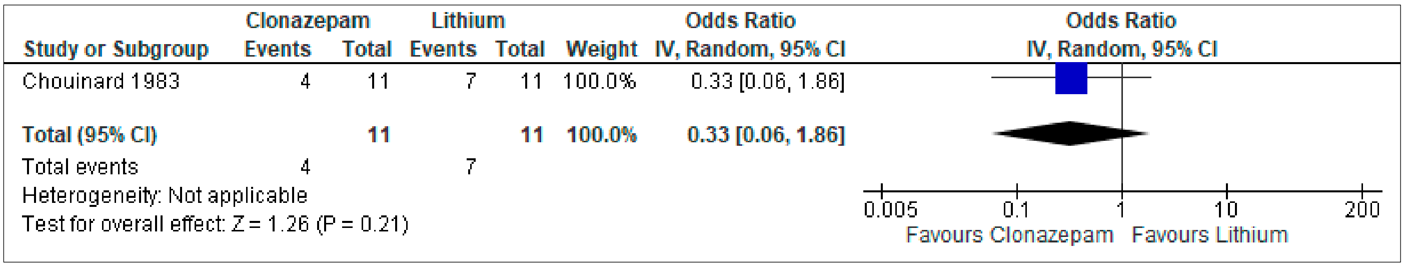

Figure S21: Clonazepam vs. Lithium. Outcome: proportion of patients prescribed as required antipsychotic medication in the acute phase of treatment (dichotomous, Haloperidol, endpoint: 10 days).

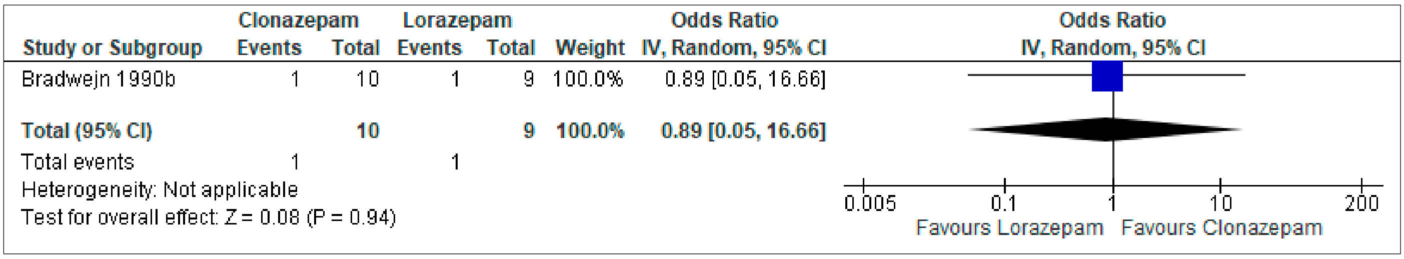

Figure S22: Clonazepam vs. Lorazepam. Outcome: proportion of patients prescribed as required antipsychotic medication over the course of treatment (dichotomous, Haloperidol, endpoint: 28 days).

Study or Subgroup	Clonazepam Mean	SD	Total	Haloperidol Mean	SD	Total	Weight	Std. Mean Difference IV, Random, 95% CI
**4.7.1 Acute**								
Yang 2009a	76.3	7.3	30	68	17.1	30	100.0%	0.62 [0.10, 1.14]
**Subtotal (95% CI)**			**30**			**30**	**100.0%**	**0.62 [0.10, 1.14]**
Heterogeneity: Not applicable								
Test for overall effect: Z = 2.35 (P = 0.02)								
**Total (95% CI)**			**30**			**30**	**100.0%**	**0.62 [0.10, 1.14]**
Heterogeneity: Not applicable								
Test for overall effect: Z = 2.35 (P = 0.02)								
Test for subgroup differences: Not applicable								

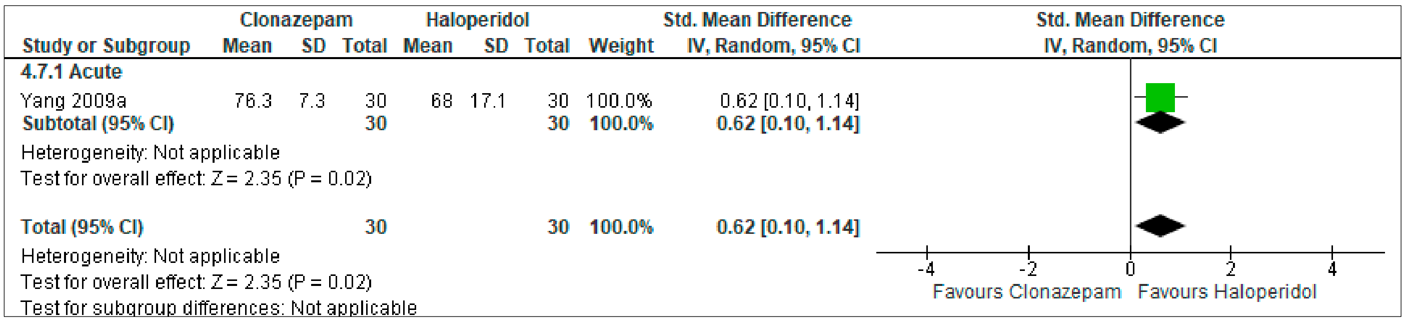

Study or Subgroup	Clonazepam Mean	SD	Total	Haloperidol Mean	SD	Total	Weight	Std. Mean Difference IV, Random, 95% CI
**4.7.2 Long-term**								
Yang 2009b	78.2	3.9	30	76.5	6.9	29	100.0%	0.30 [-0.21, 0.81]
**Subtotal (95% CI)**			**30**			**29**	**100.0%**	**0.30 [-0.21, 0.81]**
Heterogeneity: Not applicable								
Test for overall effect: Z = 1.15 (P = 0.25)								
**Total (95% CI)**			**30**			**29**	**100.0%**	**0.30 [-0.21, 0.81]**
Heterogeneity: Not applicable								
Test for overall effect: Z = 1.15 (P = 0.25)								
Test for subgroup differences: Not applicable								

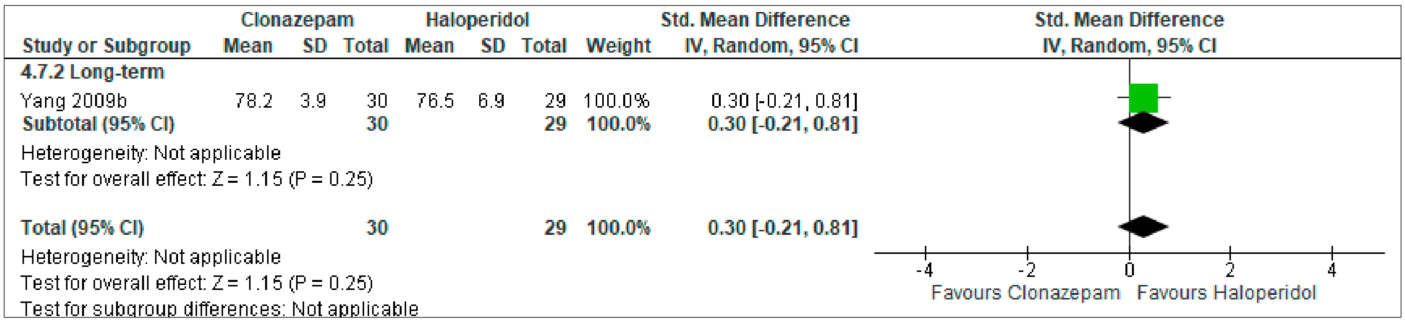

Figure S23: Clonazepam vs. Haloperidol, as add on to Valproate. Outcome: Valproate plasma levels. Yang 2009a: presents the acute effects (up to 3 weeks). Yang 2009b: presents the long-term effects (more than 3 weeks of continuous treatment).

Study or Subgroup	Clonazepam Events	Clonazepam Total	Control Events	Control Total	Weight	Odds Ratio IV, Random, 95% CI
**7.3.1 Clonazepam vs. Lorazepam**						
Bradwejn 1990a	6	11	2	13	42.9%	6.60 [0.97, 44.93]
**Subtotal (95% CI)**		**11**		**13**	**42.9%**	**6.60 [0.97, 44.93]**
Total events	6		2			
Heterogeneity: Not applicable						
Test for overall effect: Z = 1.93 (P = 0.05)						
**7.3.2 Clonazepam vs. Lithium**						
Chouinard 1983	7	11	0	11	29.9%	38.33 [1.79, 820.13]
**Subtotal (95% CI)**		**11**		**11**	**29.9%**	**38.33 [1.79, 820.13]**
Total events	7		0			
Heterogeneity: Not applicable						
Test for overall effect: Z = 2.33 (P = 0.02)						
**7.3.3 Clonazepam vs. Haloperidol**						
Chouinard 1993	0	8	1	8	27.2%	0.29 [0.01, 8.37]
**Subtotal (95% CI)**		**8**		**8**	**27.2%**	**0.29 [0.01, 8.37]**
Total events	0		1			
Heterogeneity: Not applicable						
Test for overall effect: Z = 0.72 (P = 0.47)						
**Total (95% CI)**		**30**		**32**	**100.0%**	**4.79 [0.45, 51.07]**
Total events	13		3			
Heterogeneity: Tau² = 2.44; Chi² = 4.52, df = 2 (P = 0.10); I² = 56%						
Test for overall effect: Z = 1.30 (P = 0.19)						
Test for subgroup differences: Chi² = 4.52, df = 2 (P = 0.10), I² = 55.7%						

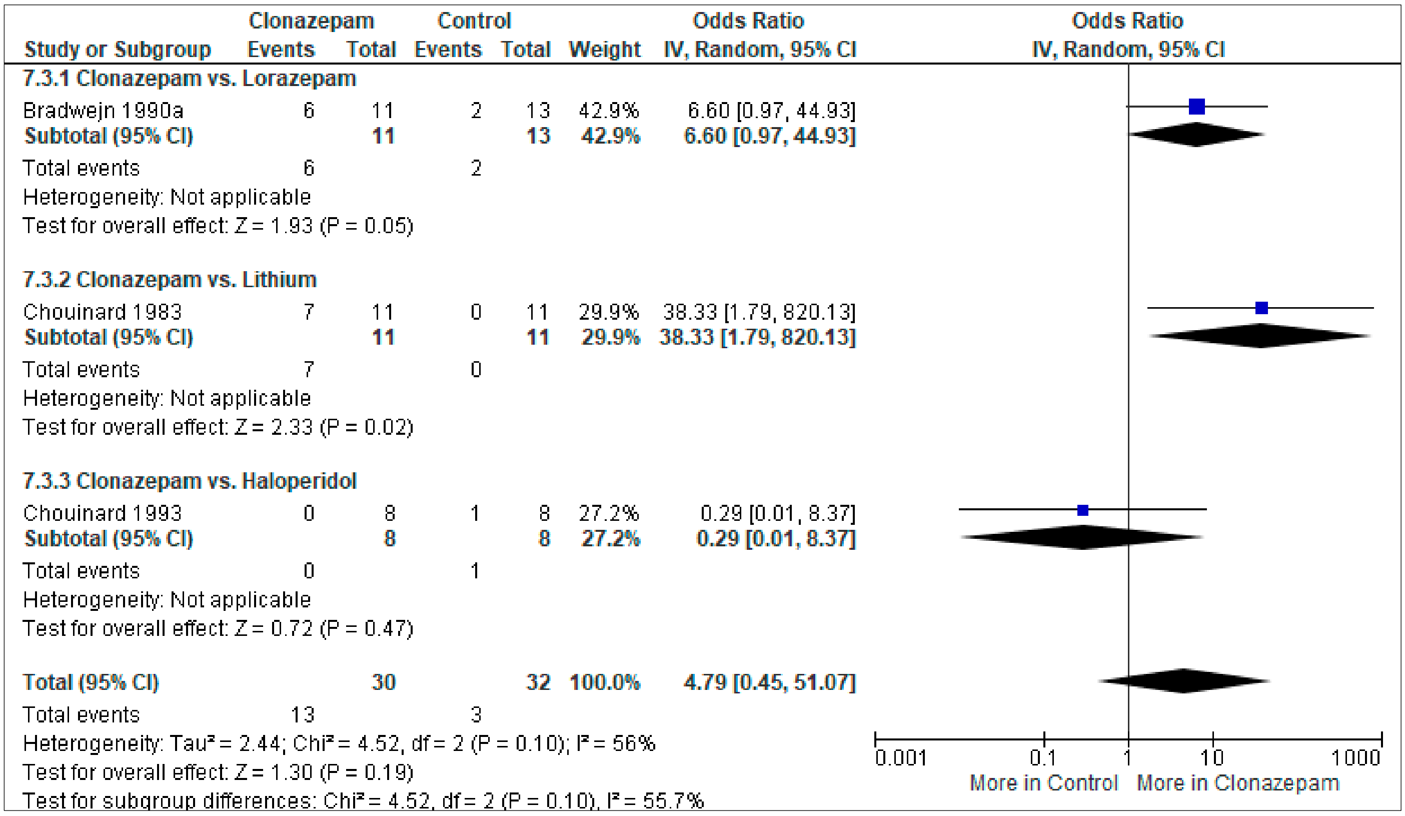

Figure S24: Clonazepam vs. any other pharmacotherapy. Outcome: sedation as a treatment-emergent adverse effect, in the acute phase of treatment (up to 3 weeks).

Study or Subgroup	Clonazepam Mean	Clonazepam SD	Clonazepam Total	Control Mean	Control SD	Control Total	Weight	Std. Mean Difference IV, Random, 95% CI
**7.4.1 Clonazepam vs. Lithium**								
Chouinard 1983	3.1	4.621	11	8.8	4.621	11	32.5%	-1.19 [-2.11, -0.27]
Clark 1997a	0.86	1.46	15	0.47	1.06	15	37.5%	0.30 [-0.42, 1.02]
**Subtotal (95% CI)**			**26**			**26**	**70.0%**	**-0.42 [-1.87, 1.04]**
Heterogeneity: Tau² = 0.92; Chi² = 6.19, df = 1 (P = 0.01); I² = 84%								
Test for overall effect: Z = 0.56 (P = 0.58)								
**7.4.2 Clonazepam vs. Haloperidol**								
Chouinard 1993	6.5	4.9	7	8.5	4.9	8	30.0%	-0.38 [-1.41, 0.64]
**Subtotal (95% CI)**			**7**			**8**	**30.0%**	**-0.38 [-1.41, 0.64]**
Heterogeneity: Not applicable								
Test for overall effect: Z = 0.73 (P = 0.46)								
**Total (95% CI)**			**33**			**34**	**100.0%**	**-0.39 [-1.29, 0.51]**
Heterogeneity: Tau² = 0.43; Chi² = 6.23, df = 2 (P = 0.04); I² = 68%								
Test for overall effect: Z = 0.85 (P = 0.40)								
Test for subgroup differences: Chi² = 0.00, df = 1 (P = 0.97), I² = 0%								

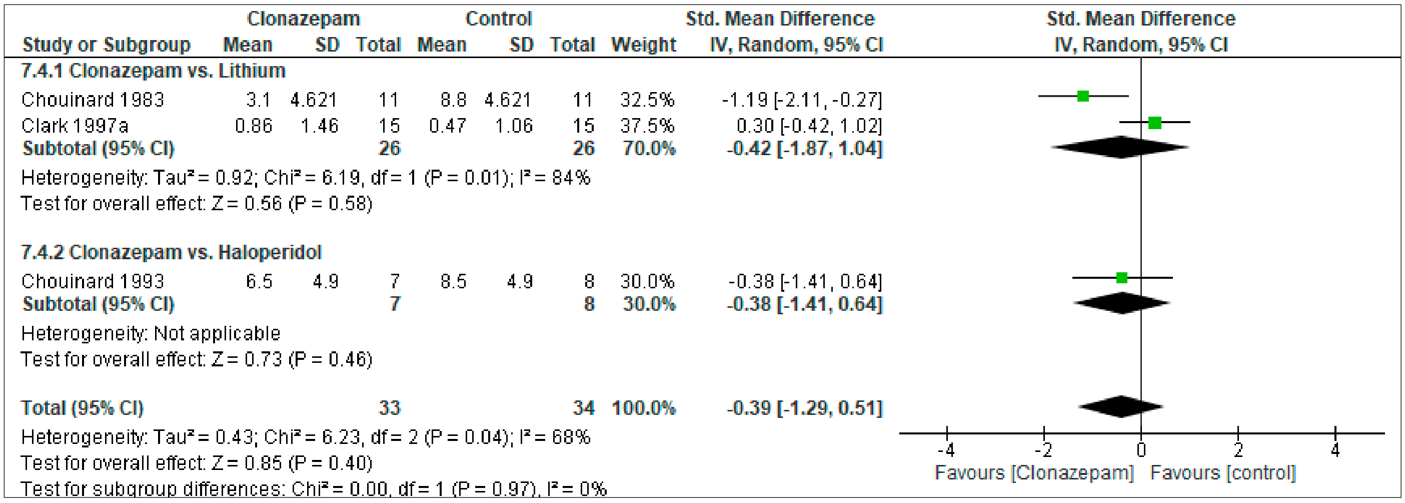

Figure S25: Clonazepam vs. any other pharmacotherapy, including placebo. Outcome: severity of extrapyramidal adverse effects (continuous).

G. Sensitivity analysis for the primary outcomes: Fixed effects model

Study or Subgroup	Clonazepam Mean	Clonazepam SD	Clonazepam Total	Placebo Mean	Placebo SD	Placebo Total	Weight	Std. Mean Difference IV, Fixed, 95% CI
Edwards 1991	-3.45	1.258	20	-1.925	1.178	20	100.0%	-1.23 [-1.91, -0.55]
**Total (95% CI)**			**20**			**20**	**100.0%**	**-1.23 [-1.91, -0.55]**
Heterogeneity: Not applicable								
Test for overall effect: Z = 3.53 (P = 0.0004)								

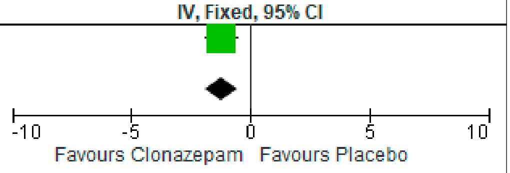

Figure S26: Clonazepam vs. Placebo. Outcome: efficacy, response to treatment (continuous). Fixed effects model.

Study or Subgroup	Favours Clonazepam Mean	Favours Clonazepam SD	Favours Clonazepam Total	Control Mean	Control SD	Control Total	Weight	Std. Mean Difference IV, Fixed, 95% CI
**2.1.1 Response - acute**								
Bradwejn 1990a	4.8	1.757	11	3.3	1.757	13	100.0%	0.82 [-0.02, 1.67]
**Subtotal (95% CI)**			**11**			**13**	**100.0%**	**0.82 [-0.02, 1.67]**
Heterogeneity: Not applicable								
Test for overall effect: Z = 1.92 (P = 0.06)								
**Total (95% CI)**			**11**			**13**	**100.0%**	**0.82 [-0.02, 1.67]**
Heterogeneity: Not applicable								
Test for overall effect: Z = 1.92 (P = 0.06)								
Test for subgroup differences: Not applicable								

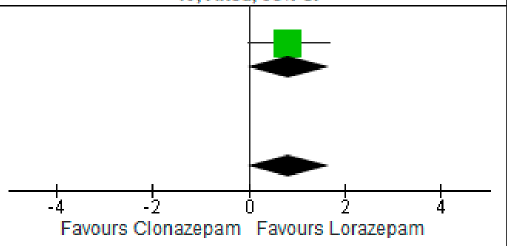

Study or Subgroup	Favours Clonazepam Mean	Favours Clonazepam SD	Favours Clonazepam Total	Control Mean	Control SD	Control Total	Weight	Std. Mean Difference IV, Fixed, 95% CI
**1 Response - acute**								
Bradwejn 1990a	4.8	1.757	11	3.3	1.757	13		0.82 [-0.02, 1.67]
			**11**			**13**	**55.8%**	
Heterogeneity: Not applicable								
Test for overall effect: Z = 1.92 (P = 0.06)								
**Total (95% CI)**			**10**			**9**	**100.0%**	**0.82 [-0.13, 1.76]**
Heterogeneity: Not applicable								
Test for overall effect: Z = 1.69 (P = 0.09)								
Test for subgroup differences: Not applicable								

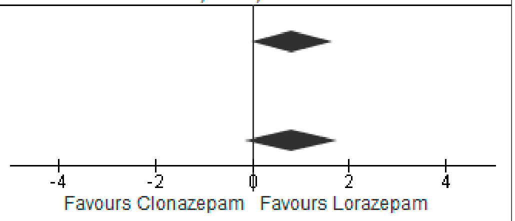

Figure S27: Clonazepam vs. Lorazepam. Outcome: efficacy, response to treatment (continuous). Bradwejn 1990a: presents the acute effects (up to 3 weeks). Bradwejn 1990b: presents the long-term effects (more than 3 weeks of continuous treatment). Fixed effects model.

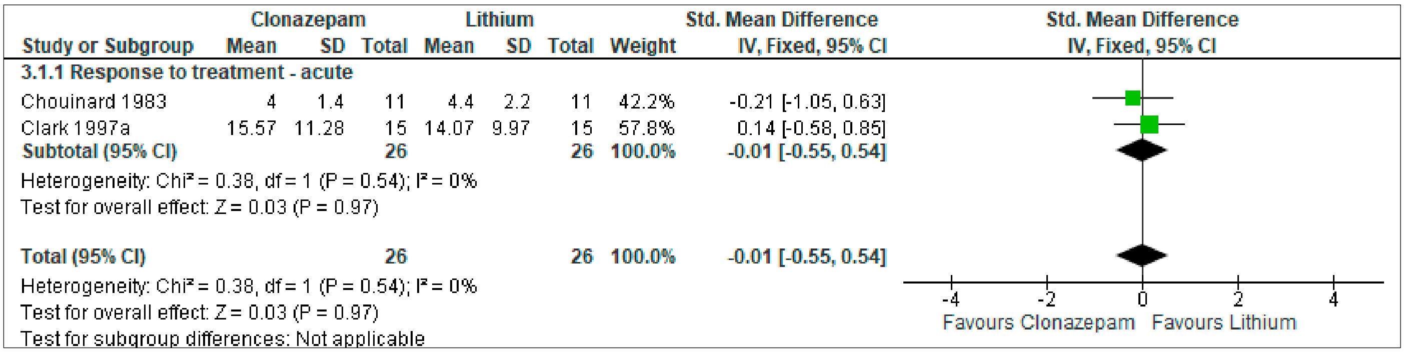

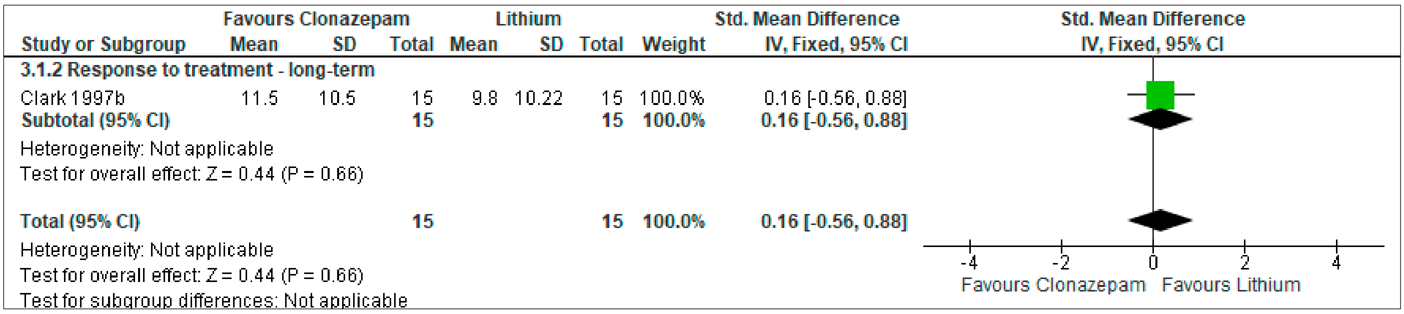

Figure S28. Clonazepam vs. Lithium. Outcome: efficacy, response to treatment (continuous). Clark 1997a: presents the acute effects (up to 3 weeks). Clark 1997b: presents the long-term effects (more than 3 weeks of continuous treatment). Fixed effects model.

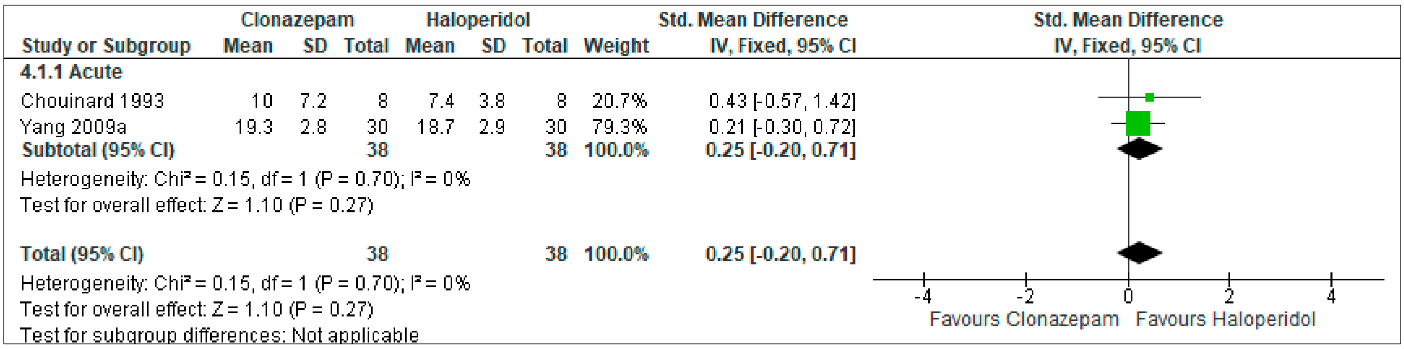

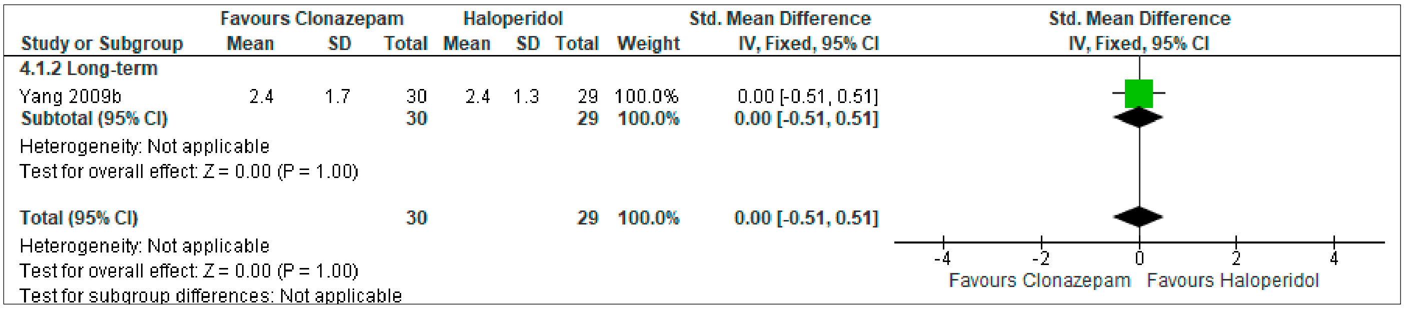

Figure S29. Clonazepam vs. Haloperidol. Outcome: efficacy, response to treatment (continuous).Yang 2009a: presents the acute effects (up to 3 weeks). Yang 2009b: presents the long-term effects (more than 3 weeks of continuous treatment). Fixed effects model.

Study or Subgroup	Clonazepam Events	Clonazepam Total	Control Events	Control Total	Weight	Odds Ratio IV, Fixed, 95% CI
**7.1.1 Clonazepam vs. placebo**						
Edwards 1991	0	20	0	20		Not estimable
**Subtotal (95% CI)**		**20**		**20**		**Not estimable**
Total events	0		0			
Heterogeneity: Not applicable						
Test for overall effect: Not applicable						
**7.1.2 Clonazepam vs. Lorazepam**						
Bradwejn 1990a	0	11	0	13		Not estimable
**Subtotal (95% CI)**		**11**		**13**		**Not estimable**
Total events	0		0			
Heterogeneity: Not applicable						
Test for overall effect: Not applicable						
**7.1.3 Clonazepam vs. Lithium**						
Chouinard 1983	0	11	0	11		Not estimable
Clark 1997a	0	15	0	15		Not estimable
**Subtotal (95% CI)**		**26**		**26**		**Not estimable**
Total events	0		0			
Heterogeneity: Not applicable						
Test for overall effect: Not applicable						
**7.1.4 Clonazepam vs. Haloperidol**						
Chouinard 1993	0	8	2	8	50.6%	0.15 [0.01, 3.77]
Yang 2009a	0	30	1	30	49.4%	0.32 [0.01, 8.24]
**Subtotal (95% CI)**		**38**		**38**	**100.0%**	**0.22 [0.02, 2.16]**
Total events	0		3			
Heterogeneity: Chi² = 0.10, df = 1 (P = 0.75); I² = 0%						
Test for overall effect: Z = 1.30 (P = 0.19)						
**Total (95% CI)**		**95**		**97**	**100.0%**	**0.22 [0.02, 2.16]**
Total events	0		3			
Heterogeneity: Chi² = 0.10, df = 1 (P = 0.75); I² = 0%						
Test for overall effect: Z = 1.30 (P = 0.19)						
Test for subgroup differences: Not applicable						

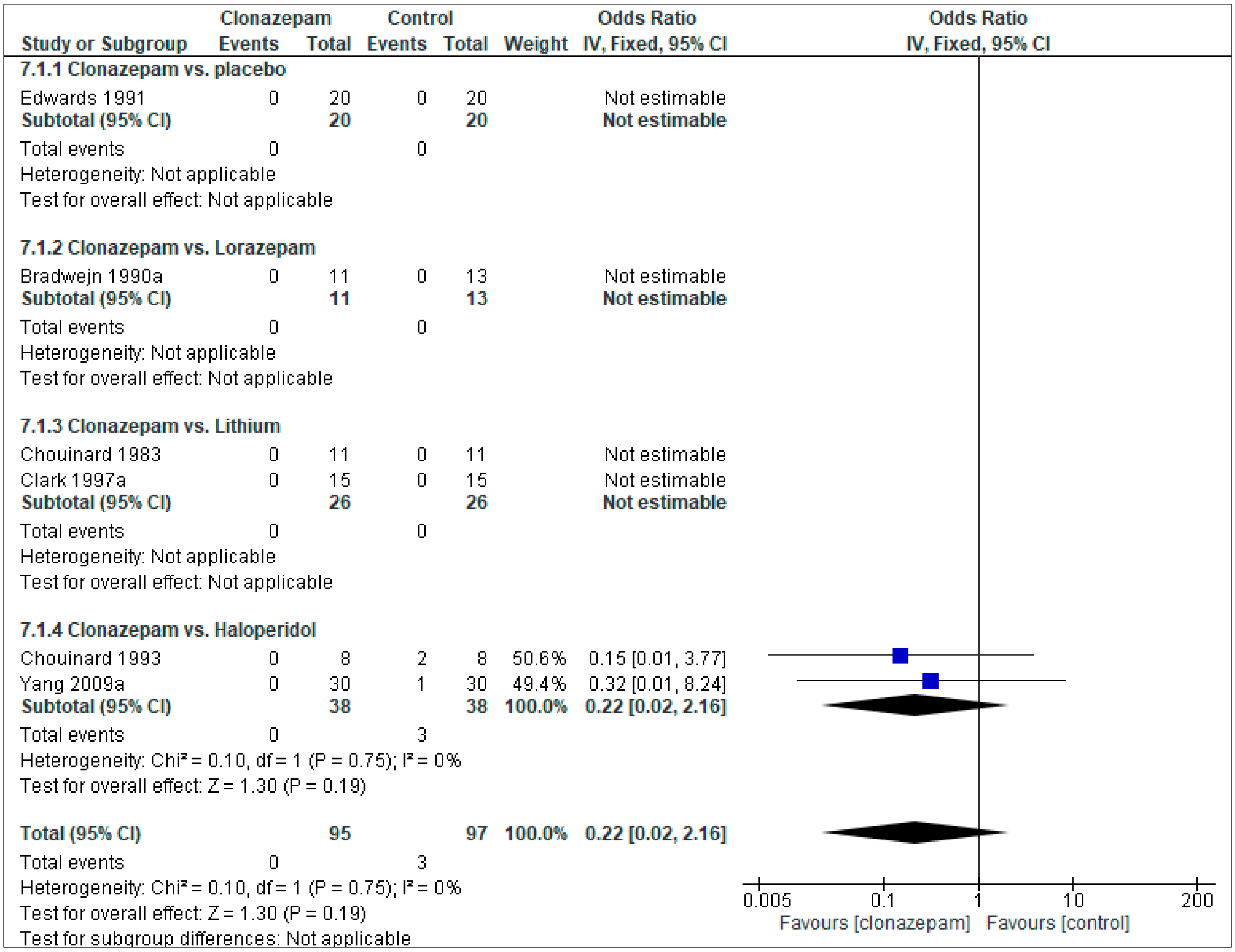

Study or Subgroup	Clonazepam Events	Clonazepam Total	Control Events	Control Total	Weight	Odds Ratio M-H, Fixed, 95% CI
Bradwejn 1990b	0	10	0	9		Not estimable
Clark 1997b	0	15	0	15		Not estimable
Yang 2009b	0	30	1	30	100.0%	0.32 [0.01, 8.24]
**Total (95% CI)**		**55**		**54**	**100.0%**	**0.32 [0.01, 8.24]**
Total events	0		1			
Heterogeneity: Not applicable						
Test for overall effect: Z = 0.68 (P = 0.49)						

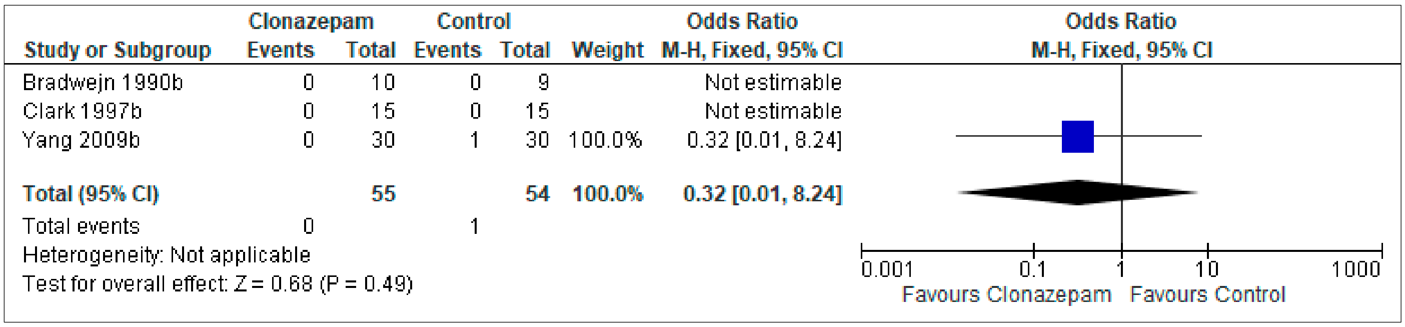

Figure S30. Tolerability (discontinuation due to adverse effects, measured as the proportion of patients who dropped out due to adverse effects): Clonazepam vs. any other pharmacotherapy, including placebo. Bradwejn 1990a, Clark 1997a and Yang 2009a: present the acute effects (up to 3 weeks). Bradwejn 1990b, Clark 1997b and Yang 2009b: present the long-term effects (more than 3 weeks of continuous treatment). Fixed effects model.

Study or Subgroup	Clonazepam Events	Clonazepam Total	Control Events	Control Total	Weight	Odds Ratio IV, Fixed, 95% CI
**6.1.1 Clonazepam vs. placebo**						
Edwards 1991	0	20	0	20		Not estimable
**Subtotal (95% CI)**		**20**		**20**		**Not estimable**
Total events	0		0			
Heterogeneity: Not applicable						
Test for overall effect: Not applicable						
**6.1.2 Clonazepam vs. Lorazepam**						
Bradwejn 1990a	1	11	2	13	35.3%	0.55 [0.04, 7.03]
**Subtotal (95% CI)**		**11**		**13**	**35.3%**	**0.55 [0.04, 7.03]**
Total events	1		2			
Heterogeneity: Not applicable						
Test for overall effect: Z = 0.46 (P = 0.65)						
**6.1.3 Clonazepam vs. Lithium**						
Chouinard 1983	0	11	1	11	21.0%	0.30 [0.01, 8.32]
Clark 1997a	1	15	0	15	21.3%	3.21 [0.12, 85.20]
**Subtotal (95% CI)**		**26**		**26**	**42.3%**	**1.00 [0.10, 10.25]**
Total events	1		1			
Heterogeneity: Chi² = 0.98, df = 1 (P = 0.32); I² = 0%						
Test for overall effect: Z = 0.00 (P = 1.00)						
**6.1.4 Clonazepam vs. Haloperidol**						
Chouinard 1993	0	8	2	8	22.4%	0.15 [0.01, 3.77]
Yang 2009a	0	30	0	30		Not estimable
**Subtotal (95% CI)**		**38**		**38**	**22.4%**	**0.15 [0.01, 3.77]**
Total events	0		2			
Heterogeneity: Not applicable						
Test for overall effect: Z = 1.15 (P = 0.25)						
**Total (95% CI)**		**95**		**97**	**100.0%**	**0.53 [0.12, 2.42]**
Total events	2		5			
Heterogeneity: Chi² = 1.84, df = 3 (P = 0.61); I² = 0%						
Test for overall effect: Z = 0.82 (P = 0.41)						
Test for subgroup differences: Chi² = 0.86, df = 2 (P = 0.65), I² = 0%						

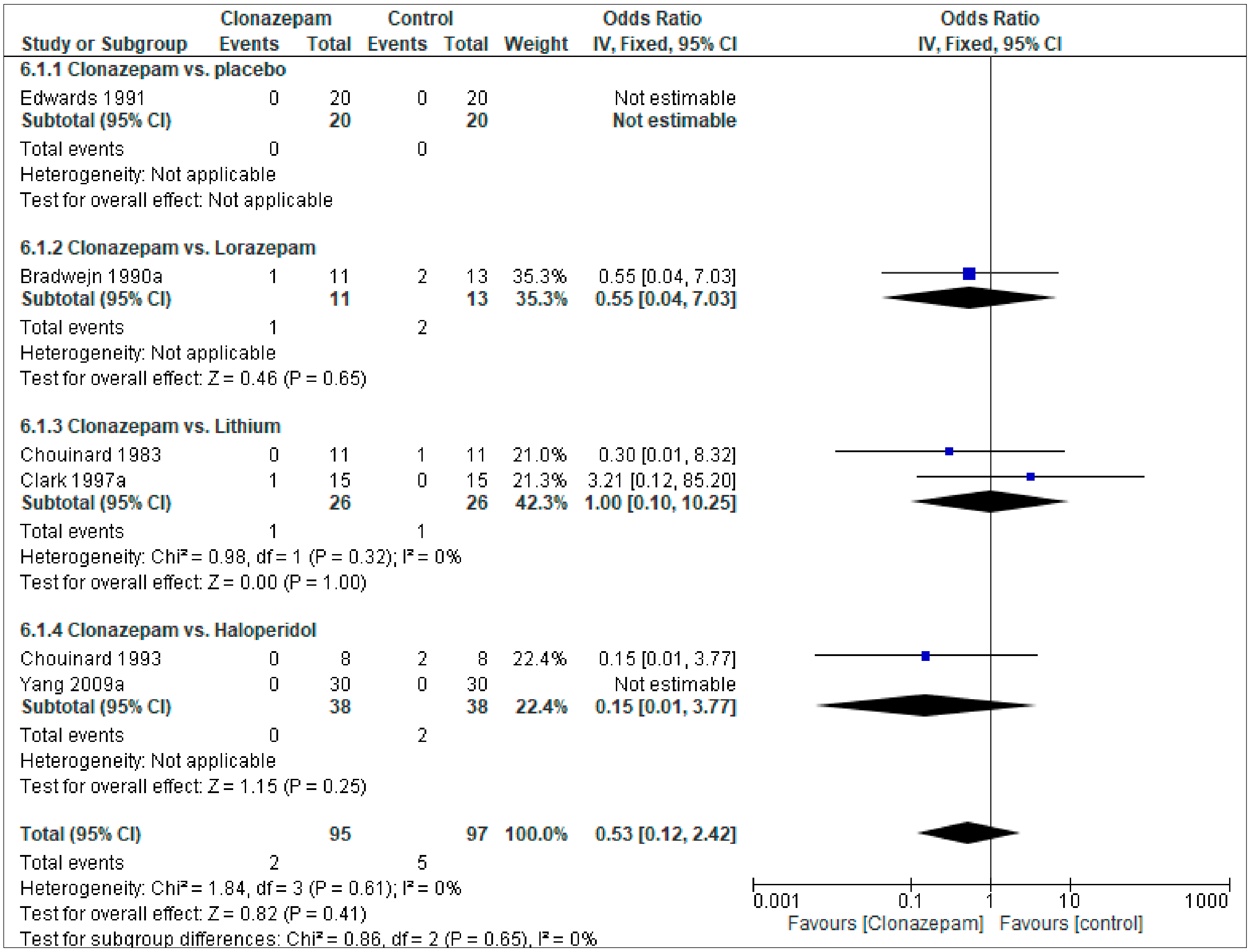

Study or Subgroup	Clonazepam Events	Clonazepam Total	Control Events	Control Total	Weight	Odds Ratio IV, Fixed, 95% CI
Bradwejn 1990b	1	10	4	9	47.0%	0.14 [0.01, 1.61]
Clark 1997b	1	15	0	15	26.2%	3.21 [0.12, 85.20]
Yang 2009b	0	30	1	30	26.8%	0.32 [0.01, 8.24]
**Total (95% CI)**		**55**		**54**	**100.0%**	**0.40 [0.07, 2.12]**
Total events	2		5			
Heterogeneity: Chi² = 2.28, df = 2 (P = 0.32); I² = 12%						
Test for overall effect: Z = 1.08 (P = 0.28)						

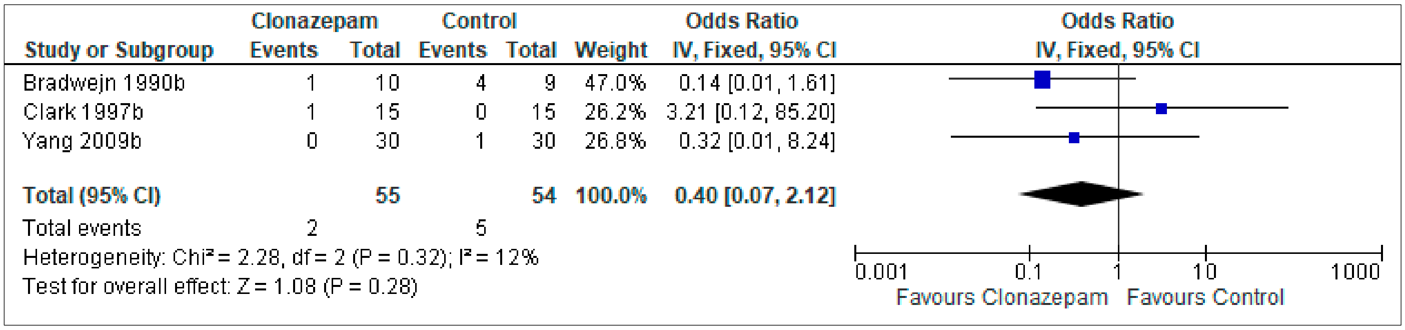

Figure S31. Acceptability (all cause discontinuation, measured as the proportion of patients who dropped out due to any reason): Clonazepam vs. any other pharmacotherapy, including placebo. Bradwejn 1990a, Clark 1997a and Yang 2009a: present the acute effects (up to 3 weeks). Bradwejn 1990b, Clark 1997b and Yang 2009b: present the medium and long-term effects (more than 3 weeks of continuous treatment). Fixed effects model.
